# Light has a specific role in modulating *Arabidopsis *gene expression at low temperature

**DOI:** 10.1186/1471-2229-8-13

**Published:** 2008-01-29

**Authors:** Arto J Soitamo, Mirva Piippo, Yagut Allahverdiyeva, Natalia Battchikova, Eva-Mari Aro

**Affiliations:** 1 University of Turku, Department of Biology, Plant Physiology and Molecular Biology, Tykistokatu 6, BioCity A, 6^th ^floor, FIN-20520 Turku, Finland

## Abstract

**Background:**

Light and temperature are the key abiotic modulators of plant gene expression. In the present work the effect of light under low temperature treatment was analyzed by using microarrays. Specific attention was paid to the up and down regulated genes by using promoter analysis. This approach revealed putative regulatory networks of transcription factors behind the induction or repression of the genes.

**Results:**

Induction of a few oxidative stress related genes occurred only under the Cold/Light treatment including genes encoding iron superoxide dismutase (*FeSOD*) and glutathione-dependent hydrogen peroxide peroxidases (*GPX*). The ascorbate dependent water-water cycle genes showed no response to Cold/Light or Cold/Dark treatments. Cold/Light specifically induced genes encoding protective molecules like phenylpropanoids and photosynthesis-related carotenoids also involved in the biosynthesis of hormone abscisic acid (ABA) crucial for cold acclimation. The enhanced/repressed transcript levels were not always reflected on the respective protein levels as demonstrated by dehydrin proteins.

**Conclusion:**

Cold/Light up regulated twice as many genes as the Cold/Dark treatment and only the combination of light and low temperature enhanced the expression of several genes earlier described as cold-responsive genes. Cold/Light-induced genes included both cold-responsive transcription factors and several novel ones containing zinc-finger, MYB, NAC and AP2 domains. These are likely to function in concert in enhancing gene expression. Similar response elements were found in the promoter regions of both the transcription factors and their target genes implying a possible parallel regulation or amplification of the environmental signals according to the metabolic/redox state in the cells.

## Backround

Light has a pronounced effect on gene expression via photoreceptors [1] particularly during the early photomorphogenetic development of plants. Light is also a driving force for photosynthesis, which in turn regulates many metabolic processes in cells. Such regulation occurs either directly by production of ATP and reducing power NADPH or indirectly e.g. via redox active compounds, like thioredoxins and glutathione (GSH), which then might exert an effect on gene expression [2]. Transcription of nuclear genes is also known to be orchestrated by photosynthesis products [3,4].

A wealth of global gene expression data is now available from *Arabidopsis *plants exposed to various light treatments as well as to low temperatures and salt or dehydration treatments [5-10]. Gene transcription, regulated by a number of transcription factors, is strongly influenced both by abiotic environmental factors and various cellular compounds [7,11-15]. Although in some recent experiments a specific role of light has been implicated in response of plants to biotic stress [11,16], the role of light in global gene expression analysis, particularly when combined with various other abiotic stress conditions, has remained elusive. Indeed, besides its function via photoreceptors, light exerts effects on gene expression also via the photosynthetic apparatus, whose function can be strongly modulated by various environmental stress conditions [17]. Light and temperature changes in natural environments often occur in parallel but the dissection of the role of light and the function of the photosynthetic apparatus, from the sole low temperature effect have been studied only with a limited set of genes [18,19].

*Arabidopsis *is a freezing tolerant plant and it's cold tolerance increases upon exposure of plants to low temperature [20]. Moreover, during the cold acclimation process light is required for enhanced freezing tolerance in *Arabidopsis *leaves [21]. Here, we have performed transcript profiling of *Arabidopsis thaliana *leaves after a low temperature treatment of plants in light or in darkness or after a sole light or dark treatment. Light had a profound effect in increasing the amount of transcripts from so-called cold-responsive genes. More importantly, the condition of cold and light induced a specific set of genes, which apparently are important in the development of freezing tolerance. The complexity of gene expression patterns is emphasized by the fact that more than 40 differentially regulated transcription factors were found. The regulatory role of these transcription factors and their target genes for the development of *Arabidopsis *cold acclimation is discussed.

## Results

### Physiological consequences of the cold treatments on *Arabidopsis *photosynthetic apparatus

Eight-week old *Arabidopsis *plants were transferred from normal growth temperature (23°C, 60% relative humidity) directly to low temperature (3°C, 60% relative humidity) under normal growth light (100 μmol photons m^-2 ^s^-1^) or to darkness for eight hours. About 10% decrease in the photochemical efficiency (Fv/Fm) and the oxygen evolving activity of photosystem II (PSII) was measured after cold and light (hereafter, Cold/Light or C/L) treatment, but not after cold and dark (hereafter Cold/Dark or C/D) treatment (Table 1). In our Nordic consortium project (NKJ), parallel experiments showed about 2.5 times more severe loss in the activity of PSI after the Cold/Light treatment [22], which implies nearly 30% inhibition of PSI in our Cold/Light treatment.

**Table 1:** Effect of cold treatments on functional properties of PSII

**Treatment**	Fv/Fm	% of Control	O_2_-evolution μmol O_2 _mg Chl^-1 ^h^-1^	% of Control
Light Control	0.81 ± 0.01	100	198 ± 14	100
Cold/Light	0.71 ± 0.01	88	175 ± 13	88
Dark Control	0.79 ± 0.02	97	139 ± 12	100
Cold/Dark	0.80 ± 0.01	98	143 ± 15	103

The values are the mean from 6 (Fv/Fm) and 8 (O_2_-evolution) independent experiments ± SD.

**Figure 1:**
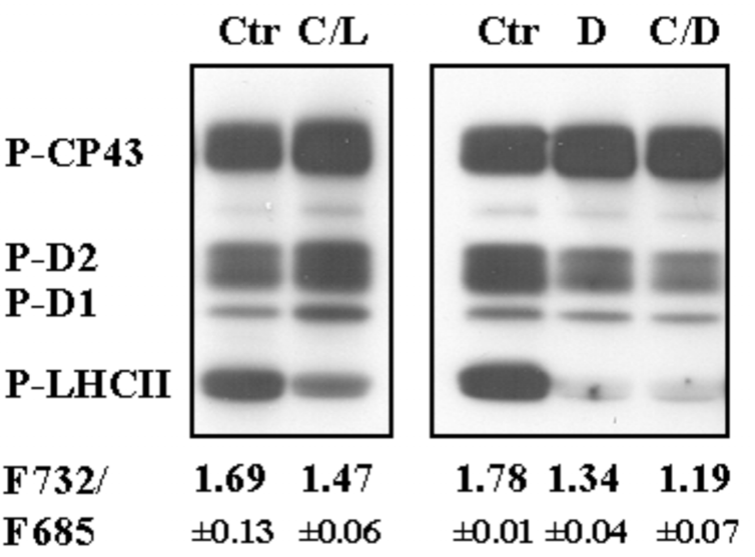
**Phosphothreonine-immunoblot of thylakoid proteins isolated from *Arabidopsis *leaves after four different treatments**: Control (Ctr), Cold/Light (C/L), Dark (D) and Cold/Dark (C/D). Below the immunoblot, 77 K fluorescence emission ratios (F732/F685 ± S.D.) of thylakoids from differentially treated plants are given. F732 stands for the fluorescence peak at 732 nm representing the emission from PSI and F685 for the fluorescence peak at 685 nm from PSII. Differences in F732/F685 ratios are related to reversible phosphorylation of the light-harvesting chl a/b proteins (LHCII) and their attachment with PSI (phosphorylated, high ratio) and PSII (non-phosphorylated, low ratio). P-CP43, P-D2, P-D1 denote the phosphorylated proteins of PSII core, P-LHCII denote the LHCII phosphoproteins.

The redox state of thylakoid proteins in chloroplasts were monitored by preparing a phosphothreonine-immunoblot from differentially treated *Arabidopsis *leaves (Figure 1). The amount of phoshorylated PSII core proteins (P-CP43, P-D2, P-D1) increased under Cold/Light condition indicating an increased reduction state of the plastoquinone pool (PQ pool) between PSII and PSI [23]. On the other hand, LHCII proteins were partially dephosphorylated under Cold/Light condition and completely dephosphorylated after Cold/Dark and Dark treatments. In addition, 77 K fluorescence measurements, demonstrating the proportional amount of LHCII proteins attached to either PSI (F732) or PSII (F685), indicated that the proportion of LHCII proteins attached to PSII (F685) increased under Cold/Light and even more under Cold/Dark conditions (Figure 1, at the bottom). This reflected changes in the redox state of chloroplast stroma as well as in the components of the electron transport chain. Upon accumulation of reduced thiols in the stroma under the Cold/Light condition resulted in the inhibition of the LHCII kinase, whereas in darkness the LHCII kinase was deactivated due to the oxidation of the electron transfer chain (as well as the stroma) [23].

### Overview of gene expression changes in Cold/Light, Cold/Dark and Eight-Hour Dark treatments

The cDNA microarray experiments are based on the *Arabidopsis *GEM1 clone set purchased from InCyte Genomics, Palo Alto, CA, USA consisting of circa 8000 ESTs corresponding about 6500 unique genes [4]. It is important to note that this cDNA microarray is containing only one third of annotated genes present in whole *Arabidopsis *genome. The microarray experiments were designed so that all treatments were compared to the control plants harvested from the controlled-environment chambers (100 μmol photons m^-2 ^s^-1^, 23°C) at the same hour of the day as the treated plants were harvested; thus making the light and low temperature treatments comparable with each other with respect to the circadian effects on gene expression.

**Figure 2:**
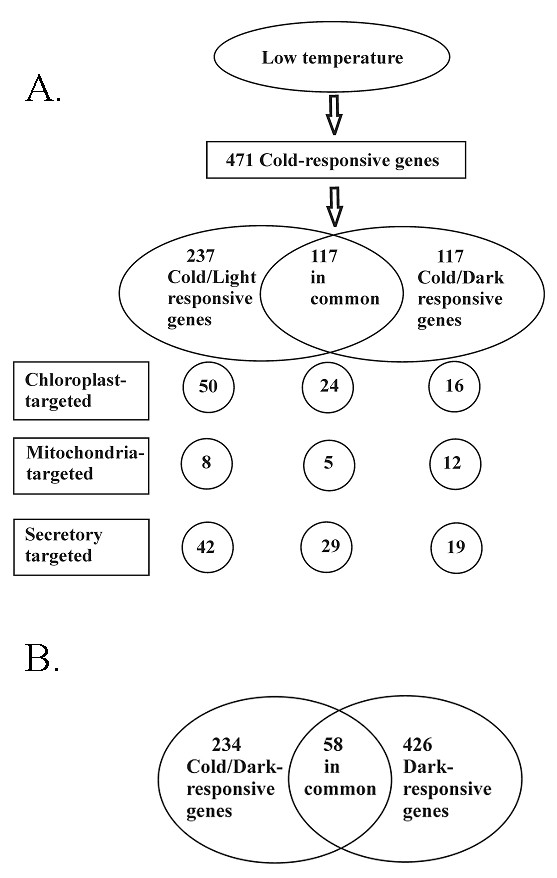
**Summary of gene expression data after three different treatments: Cold/Light, Cold/Dark and Dark treatments**. **A**. Number of genes showing at least two-fold up or down regulation after the Cold/Light and Cold/Dark treatments. The predicted localization of gene products (TargetP program) is indicated in the lower part of the Figure. **B**. A Venn diagram indicating the number of genes showing at least two-fold up or down regulation after the Cold/Dark and Dark treatments.

For defining the up or down regulation of the gene, we used two-fold expression changes as a cut off value (treated plants compared to control plants) and the Students t-test for determining statistical significance of each gene in different treatments (p-value less than 0,05 including false discovery rate (FDR)). As a result, 471 cold-responsive genes were obtained (Figure 2A and Additional file 1), of which only 117 were common for both the Cold/Light and Cold/Dark treatments. Many of these genes were established cold-responsive genes. In addition, there were 237 genes responding only to the Cold/Light treatment and 117 genes responding only to the Cold/Dark treatment. As a control to the Cold/Dark treatment, it was necessary to find out how the eight-hour darkness under normal growth temperature (hereafter Dark or D) modulates the gene expression. As depicted in Figure 2B, 234 genes were considered as Cold/Dark responsive genes, but even higher number of genes (426) turned out as only the dark-responsive genes. Of these, only 58 genes were regulated similarly in both dark treatments.

**Figure 3:**
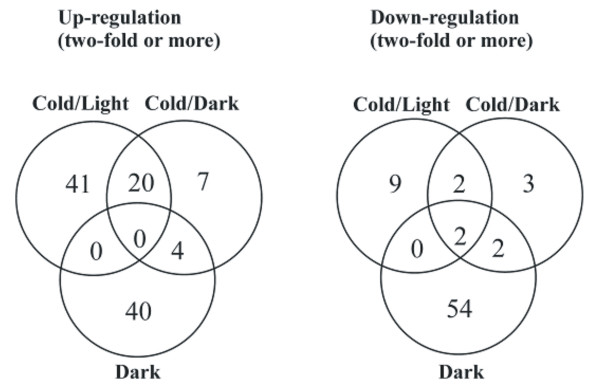
**Response of genes encoding chloroplast-targeted proteins to the Cold/Light, Cold/Dark and Dark treatments**. Venn diagram indicating differential expression of genes upon the three different treatments.

Cold-responsive genes were also analyzed with respect to possible organelle-targeting signals (Figures 2A and 3). Cold/Light induced 61 and repressed 13 genes with chloroplast-targeting signal, as predicted by TargetP [24]. Of these, 41 and 9 genes, respectively, responded specifically only in the Cold/Light treatment (Figure 3). Indeed, here were only a few Cold/Dark specific genes with chloroplast targeting signal. The eight-hour dark treatment at 23°C, on the other hand, modified the expression of a large number of genes encoding chloroplast-targeted proteins; 40 were up regulated and 54 down regulated.

### Expression of established cold responsive genes in Cold/Light and Cold/Dark conditions

An up regulation of many well-characterized cold-responsive genes was found upon a transfer of plants from normal growth temperature to low temperature implying an initiation of the cold acclimation/dehydration process. The expression of several canonical cold-responsive genes was more up regulated in Cold/Light than in Cold/Dark condition (Table 2 and Additional files 2 and 4). These included genes encoding the low temperature-induced proteins (LTIs), like XERO2/LTI30 (At3g50970), LTI78/RD29A (At5g52310), ERD10 (Early Response to Dehydration, At1g20450), ERD3 (At4g19120), KIN1 (At5g15960), two galactinol synthases (At1g56600 and At1g09350) and dehydrin RAB18 (At5g66400). Several other low temperature responsive genes were also found but their expression did not differ whether the low temperature treatment was given in light or in darkness.

**Table 2:** Up or down regulated transcripts upon different temperature and light treatments

AGI-code and Description **Cold and Dehydration Responsive Genes**	**Control**	**Cold/Light**	**Cold/Dark**	**Dark**
At1g09350 galactinol synthase, AtGolS3	0.9 ± 0.1	42.0 ± 3.0*	**17.1 ± 1.5**	1.0 ± 0.4
At3g50970 dehydrin (XERO2) (Low-temperature-induced protein, LTI30)	1.0 ± 0.1	**17.8 ± 3.0**	**11.7 ± 5.3**	**0.4 ± 0.1**
At5g52310 low-temperature-induced protein 78, (RD29A) **(UP)**	1.3 ± 0.2	11.6 ± 3.5* ^(a)^	**3.6 ± 1.7**	**0.1 ± 0.1**
At1g20450 dehydrin (ERD10, Low-temperature-induced protein, LTI45)	1.3 ± 0.2	10.4 ± 1.4*	**3.8 ± 0.6**	**0.6 ± 0.1**
At4g19120 ERD3 protein	1.3 ± 0.2	3.5 ± 0.6*	1.6 ± 0.4	**0.4 ± 0.1**
At5g15960 stress-induced protein KIN1 **(UP)**	1.3 ± 0.1	**2.6 ± 0.9**	1.7 ± 0.2	0.1 ± 0.1
At1g56600 galactinol synthase, AtGolS2 **(Down)**	1.2 ± 0.2	2.1 ± 0.2*	1.2 ± 0.3	0.7 ± 0.2
At5g55400 dehydrin RAB18	1.2 ± 0.1	**2.0 ± 0.3**	0.9 ± 1.0	0.5 ± 0.3
**LHCB genes**				
At3g27690 light harvesting chlorophyll A/B binding protein, LHCB 2.4 **(Down)**	1.2 ± 0.2	15.8 ± 4.1*	**1.7 ± 0.2**	0.5 ± 0.3
At2g05070 light-harvesting chlorophyll A/B binding protein, LHCB 2.2 **(Down)**	1.2 ± 0.1	8.1 ± 2.3*	1.3 ± 0.2	0.6 ± 0.3
At3g08940 chlorophyll a/b-binding protein, LHCB 4.2 **(Down)**	1.2 ± 0.1	2.7 ± 0.8*	0.7 ± 0.2	**1.7 ± 0.2**
At3g22840 early light-induced protein, ELIP1 **(Up)**	1.0 ± 0.1	2.4 ± 0.3*	1.1 ± 0.1	0.8 ± 0.2
At1g29930 light harvesting chlorophyll A/B binding protein **(Down)**	1.1 ± 0.1	2.1 ± 0.3*	0.9 ± 0.1	1.2 ± 0.2
**Photosystem I related genes**				
At5g64040 photosystem I reaction center subunit, PSI-N **(Down)**	1.2 ± 0.2	2.5 ± 0.3*	1.3 ± 0.2	0.8 ± 0.1
At1g08570 thioredoxin	1.1 ± 0.1	**2.0 ± 0.2**	**1.7 ± 0.2**	**1.7 ± 0.2**
**Genes encoding chloroplast targeted proteases**				
At1g49630 Zn metalloprotease	1.2 ± 0.1	5.5 ± 0.9*	**1.9 ± 0.2**	**0.6 ± 0.1**
At5g42270 FTSH protease (H5) **(Up)**	1.1 ± 0.1	2.9 ± 0.4*	1.0 ± 0.2	**0.6 ± 0.1**
At1g50250 FTSH protease (H1) **(Up)**	1.2 ± 0.1	2.3 ± 0.3*	1.2 ± 0.2	**0.6 ± 0.1**
At1g06430 FTSH protease (H8)	1.1 ± 0.1	2.1 ± 0.3*	0.9 ± 0.2	0.7 ± 0.1
At1g09130 ATP-dependent CLP protease (CLPR3)**(-)**	1.1 ± 0.1	**2.1 ± 0.5**	1.7 ± 0.7	**1.3 ± 0.1**
At5g51070 ATP-dependent CLP protease (CLPD), ERD1 protein **(-)**	1.1 ± 0.1	**0.4 ± 0.1**	**0.5 ± 0.1**	**1.5 ± 0.1**
**Genes Encoding Chloroplast Targeted ROS Scavenging Enzymes**				
At4g11600 glutathione peroxidise **(Up)**	1.3 ± 0.1	5.7 ± 1.1*	1.4 ± 0.2	**0.7 ± 0.1**
At4g25100 iron superoxide dismutase (FeSOD) **(Up)**	1.2 ± 0.3	3.2 ± 0.4*	**1.7 ± 0.2**	**1.8 ± 0.3**
At2g25080 glutathione peroxidise **(Down)**	1.3 ± 0.2	2.8 ± 0.3*	1.4 ± 0.3	1.4 ± 0.2
At3g54660 gluthatione reductase **(-)**	1.2 ± 0.1	2.1 ± 0.2*	**1.4 ± 0.1**	**0.6 ± 0.1**
**Expression of Catalase and Ascorbate Reductase Genes**				
At4g35090 catalase 2 **(Up)**	1.3 ± 0.1	**6.4 ± 1.4**	**3.8 ± 1.2**	**2.5 ± 0.4**
At3g09940 monodehydroascorbate reductase	0.9 ± 0.1	0.9 ± 0.1	1.2 ± 0.2	0.8 ± 0.1
At3g52880 monodehydroascorbate reductase	1.3 ± 0.1	0.9 ± 0.2	**0.7 ± 0.1**	0.7 ± 0.1
At1g20630 catalase 1 **(Down)**	1.1 ± 0.1	0.8 ± 0.1	0.9 ± 0.2	1.2 ± 0.2
At1g75270 dehydroascorbate reductase	0.9 ± 0.1	**0.6 ± 0.1**	**0.6 ± 0.1**	0.5 ± 0.2
At5g03630 monodehydroascorbate reductase	1.3 ± 0.1	**0.5 ± 0.1**	**0.6 ± 0.1**	0.9 ± 0.2
At1g19570 dehydroascorbate reductase	0.9 ± 0.2	0.4 ± 0.2	**0.4 ± 0.1**	**0.3 ± 0.1**
At1g20620 catalase 3 **(Down)**	1.5 ± 0.3	**0.4 ± 0.1**	**0.5 ± 0.1**	**2.4 ± 0.2**
**Carotenoid Biosynthesis Genes**				
At5g67030 zeaxanthin epoxidase precursor, (LOS6/ABA1)(ZEP)	1.3 ± 0.2	3.8 ± 0.6*	1.7 ± 0.5	0.7 ± 0.1
At1g74470 geranylgeranyl reductase	1.4 ± 0.1	3.0 ± 0.4*	1.1 ± 0.1	1.3 ± 0.2
At4g32770 tocopherol cyclase (SXD1)	1.0 ± 0.1	2.1 ± 0.2*	1.0 ± 0.1	0.8 ± 0.2
At1g08550 violaxanthin de-epoxidase precursor, (NPQ1)	1.2 ± 0.1	0.9 ± 0.1	**0.7 ± 0.1**	**0.6 ± 0.1**
**Chlorophyll Biosynthesis Genes**				
At1g58290 glutamyl-tRNA reductase 1 (GluTR) (HEMA1)	1.0 ± 0.1	6.5 ± 0.7*	2.6 ± 0.9	1.2 ± 0.2
At3g56940 dicarboxylate diiron protein, (CHL27, CRD1)	1.2 ± 0.1	4.5 ± 0.6*	**1.4 ± 0.1**	1.1 ± 0.2
At5g13630 Mg-chelatase H-subunit (CHLH)	1.2 ± 0.1	2.2 ± 0.2*	1.2 ± 0.2	0.9 ± 0.1
**Phenylpropanoid Pathway Genes**				
At5g17050 UDP glucose:flavonoid 3-o-glucosyl-transferase	1.0 ± 0.1	12.6 ± 3.7*	1.9 ± 0.8	0.8 ± 0.1
At3g53260 phenylalanine ammonia-lyase (PAL2)**(-)**	1.2 ± 0.2	**3.7 ± 0.6**	1.9 ± 0.7	1.2 ± 0.1
At5g13930 chalcone synthase (naringenin-chalcone synthase) **(Up)**	1.0 ± 0.1	**2.9 ± 1.0**	1.0 ± 0.2	0.6 ± 0.4
At4g30210 NADPH-cytochrome p450 reductase, (ATR2)	1.2 ± 0.1	**2.5 ± 0.2**	**2.1 ± 0.3**	1.3 ± 0.5
At1g15950 cinnamoyl-CoA reductase	1.0 ± 0.1	2.5 ± 0.2*	1.4 ± 0.3	0.9 ± 0.2
At4g34050 caffeoyl-CoA 3-O-methyltransferase	1.1 ± 0.1	**2.2 ± 0.5**	1.1 ± 0.2	0.6 ± 0.1
**Carbon metabolism genes**				
At1g32900 starch synthase	1.3 ± 0.4	**6.1 ± 2.1**	4.0 ± 3.2	1.1 ± 0.2
At4g17090 glycosyl hydrolase family 14 (beta-amylase)	1.2 ± 0.1	**6.1 ± 0.7**	3.2 ± 1.4	**0.5 ± 0.2**
At1g08920 sugar transporter, putative similar to ERD6 protein	1.0 ± 0.1	**3.4 ± 0.8**	**2.2 ± 0.3**	0.8 ± 0.1
At3g01550 triose/phosphate translocator	1.1 ± 0.1	2.4 ± 0.2*	1.3 ± 0.2	1.0 ± 0.1
At4g38970 plastidic fructose-bisphosphate aldolase **(UP)**	1.3 ± 0.2	**2.0 ± 0.2**	2.4 ± 0.4	0.3 ± 0.2
At1g69830 alpha-amylase (1,4-alpha-D-glucan glucanohydrolase)	1.3 ± 0.2	1.0 ± 0.2	**0.5 ± 0.1**	**0.3 ± 0.1**
At4g36670 sugar transporter	1.0 ± 0.1	0.9 ± 0.2	2.0 ± 0.5	**1.8 ± 0.2**
At3g46970 starch phosphorylase, alpha-glucan phosphorylase, H isozyme	0.9 ± 0.1	0.9 ± 0.1	**0.5 ± 0.1**	0.6 ± 0.2
At1g71880 sucrose transporter SUC1 (sucrose-proton symporter)	1.0 ± 0.1	0.8 ± 0.1	2.9 ± 0.7*	0.5 ± 0.2
**Anaerobic Carbon Metabolism Related Genes**				
At4g33070 pyruvate decarboxylase-1, (PDC1)	1.1 ± 0.1	**6.3 ± 1.3**	**3.3 ± 0.6**	0.9 ± 0.2
At1g77120 alcohol dehydrogenase, (ADH)**(-)**	0.9 ± 0.1	**2.0 ± 0.3**	**1.5 ± 0.1**	1.0 ± 0.2
At4g17260 L-lactate dehydrogenase, (LDH)	1.0 ± 0.1	**1.7 ± 0.1**	**1.8 ± 0.1**	1.0 ± 0.2

**Control **represents internal variation (technical and/or biological) of different leaf samples from growth condition. Value ± s.e. indicates expression ratio of Treatment/Control after normalization ± standard error of the mean (n = 3–4). Genes in the table are listed in decreasing expression ratios according to Cold/Light treatment in each group of genes. ^(a)^Genes that differ significantly (Students t test p-value less than 0.05) in their expression between the **Cold/Light **and **Cold/Dark **condition are marked with an asterisk (*). Values **in bold **indicate the quality control of gene expression (a statistical test of differential expression for a specific condition, Students t test p-value less than 0.05). A comparison between Cold/Light and moderate high light responsive gene expression [4] is indicated after description of the gene: **(UP)**; a gene up regulated under moderate high light, **(Down)**; a gene down regulated under moderate high light and **(-)**; no change in the gene expression under moderate high light.

### Differential expression of genes encoding proteins associated with thylakoid function

**Figure 4:**
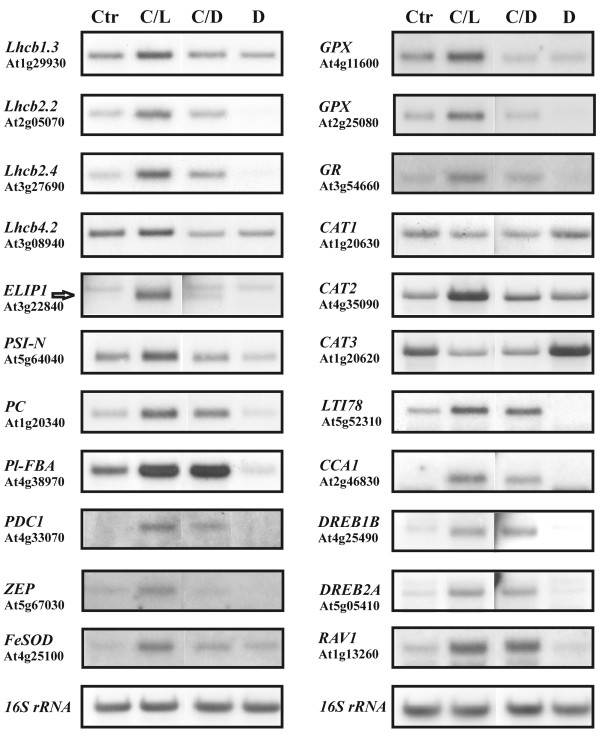
**Verification of some microarray results using northern blot analysis after four different treatments: Control (Ctr), Cold/Light (C/L), Cold/Dark (C/D) and Dark (D)**. Hybridizations were made with genes encoding: four photosystem II light harvesting proteins (LHCB) and the Early Light Inducible Protein (ELIP1); two photosystem I related (PSI) proteins, PSI-N and plastocyanin (PC); two proteins of carbohydrate metabolism, a plastidic fructose bisphoshate aldolase (Pl-FBA) and a pyruvate decarboxylase (PDC1); a ZEP protein involved in zeaxanthin and ABA biosynthesis; four chloroplast targeted proteins involved in oxygen radical scavenging and three cytoplasmic or peroxisomal catalases (CAT); a cold-responsive protein (LTI78/RD29A) and genes encoding a MYB-like (CCA1) and three AP2 transcription factors. The hybridization of the 16S rRNA probe to total RNA is shown in the bottom of the figure.

The expression of genes encoding various LHCII (LHCB) proteins was strongly enhanced under Cold/Light condition, but not under Cold/Dark condition (Table 2). On the contrary, only a few differences in the expression of nuclear genes coding for the core proteins of PSII or PSI complexes were recorded. None of the *Psb *genes coding for PSII proteins were up or down regulated more than two-fold after the Cold/Light or Cold/Dark treatment. However, there was a slight up regulation under Cold/Light condition (less than the cut off value) of *PsbW *(At2g30570) and *PsbP *(At1g77090) messages and these messages were also significantly down regulated after eight-hour dark treatment (data not shown). In addition, two genes encoding proteins closely associated with PSI, *PSI-N *(At5g64040) and thioredoxin (At1g08570) were up regulated, but only under the Cold/Light treatment (Table 2). Many of these microarray results were verified by using northern blot analysis (Figure 4).

**Figure 5:**
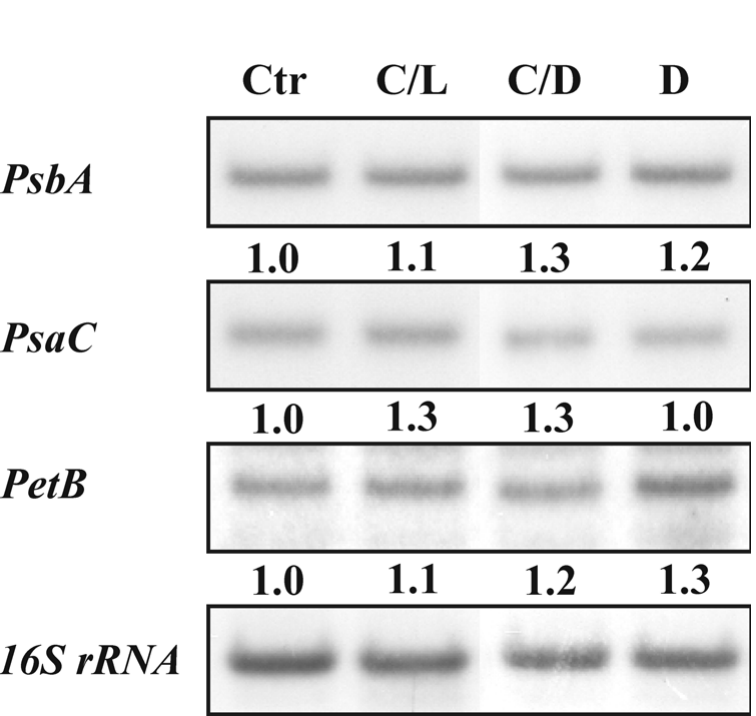
**A northern blot analysis of three chloroplast encoded transcripts (*PsbA*, *PsbC *and *PetB*) after Control (Ctr), Cold/Light (C/L), Cold/Dark (C/D) and Dark (D) treatments**. Numbers indicate the quantities of respective mRNAs after each treatment with value 1.0 for the control. Three independent northern blots were used for quantification against 16S rRNA.

We also investigated whether the experimental conditions applied here had any effect on the expression of genes encoded by the chloroplast genome (Figure 5). To this end, a northern blot analysis of *PsbA*, *PsaC *and *PetB *genes, encoding core components of PSII, PSI and the Cytb_6_f complex, respectively, was performed. However, no differential expression of these chloroplast genes was recorded between different treatments of plants.

Distinct gene expression changes were recorded for several nuclear encoded proteases, whose function is closely related to thylakoid protein complexes. Three *FTSH *genes (At5g42270, At1g50250 and At1g06430) were up regulated especially under Cold/Light condition (Table 2). These genes encode proteases involved in degradation of the D1-protein of the PSII reaction centre [25] and possibly also of the LHCB-proteins [26]. In addition, one Zn metalloprotease (At1g49630) gene was highly induced under Cold/Light condition. This gene encodes for a protease, similar to gene product of At3g19170, needed for the cleavage of the signal peptide in chloroplast and mitochondria targeted proteins [27]. Two genes encoding ATP-dependent CLP proteases were also found differentially expressed, one was up regulated (At1g09130, *ClpR3*) and the other was down regulated (At5g51070, *CLPD/ERD1*) after the Cold/Light treatment.

### Differential expression of genes related to ROS scavenging enzymes under Cold/Light, Cold/Dark and Dark conditions

**Figure 6:**
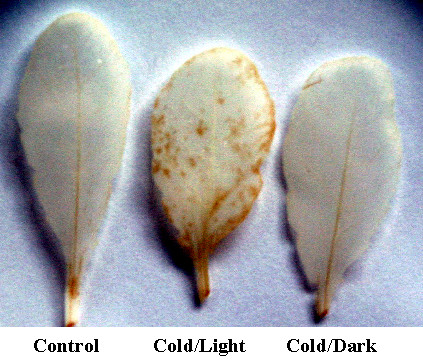
**Accumulation of oxidative stress related compounds in *Arabidopsis *leaves after Control, Cold/Light and Cold/Dark treatments**. A reddish-brown colour indicates production of oxidized DAB in leaves.

The accumulation of compounds related to oxidative stress were monitored by applying the DAB-staining method to Cold treated leaves (Figure 6). The leaves from Cold/Light treated plants revealed some reddish-brown precipitate of oxidized DAB, indicative of oxidative stress, whereas no such precipitate was detectable in Cold/Dark treated leaves. Also an induction, particularly in Cold/Light condition, was observed for a few genes encoding chloroplast-targeted enzymes active in scavenging of ROS (Table 2, Figure 4). These included an iron superoxide dismutase (*FeSOD*, At4g25100), and two glutathione dependent phosholipid hydrogen peroxide peroxidases (At4g11600 and At2g25080). The FeSOD protein seems not to have a chloroplast-targeting signal, but it has been experimentally shown to be located in the chloroplast [28]. In addition, a gene encoding chloroplast targeted glutathione reductase (*GR*) was up regulated more than two-fold under the Cold/Light condition.

It is interesting to note that the expression of genes encoding ascorbate-glutathione cycle enzymes, monodehydroascorbate reductases (*MDHAR*) and dehydroascorbate reductases (*DHAR*), located in the cytosol or in the chloroplasts, was either down regulated or unchanged (Table 2, Figure 4). Similarly, the expression of cytosolic or peroxisomal catalases were either unchanged (*CAT1*, At1g20630) or down regulated (*CAT3*, At1g20620) with catalase 2 (*CAT2*, At4g35090) as an exception, which was clearly up regulated after all three treatments i.e. under the Cold/Light, Cold/Dark and Dark conditions.

Several genes involved in the biosynthesis of photosynthesis-related isoprenoids [29-31] were also differentially expressed (Table 2). Geranylgeranyl diphosphate (GGPP) is a key compound leading to production of carotenoids, chlorophyll phytol tail, plastoquinone, phylloquinone and tocopherol (lipid-soluble compounds with antioxidant activities) [30]. The expression of geranylgeranyl reductase (*CHLP*, At1g74470), a gene encoding protein that catalyzes the hydrogenation of GGPP to phytyl diphosphate (PhyPP) and a gene encoding tocopherol cyclase (*SXD1*, At4g32770) being involved in vitamin E (tocopherol) biosynthesis [32], were significantly induced on transcript level under the Cold/Light treatment. In addition, zeaxanthine epoxidase gene (*LOS6/ABA1*, At5g67030, [33]), involved in the carotenoid pathway leading to biosynthesis of abscisic acid (ABA), was specifically up regulated (almost four-fold) only under Cold/Light condition. It is intriguing that the gene for reverse function, violaxanthine deepoxidase (*NPQ1*, At1g08550) that is important for heat dissipation of absorbed excitation energy was not up regulated under the Cold/Light condition.

Three chlorophyll biosynthesis genes were also up regulated under Cold/Light condition: glutamyl-tRNA reductase 1 (*HEMA1*, At1g58290), Mg-chelatase (*CHLH*, At5g13630) and dicarboxylate diiron protein (*CRD1*, At3g56940) (Table 2). Of these, only *HEMA1 *gene was also induced under the Cold/Dark conditions, but three times less than under Cold/Light condition.

Phenylpropanoid pathway is another complex pathway and produces phenolic compounds like flavonoids and anthocyanins that have oxidative stress alleviating abilities [34,35]. Two of differentially expressed genes encode chloroplast-targeted proteins, NADPH-ferriprotein reductase (*ATR2*, At4g30210) and UDP glucose flavonoid 3-o-glycosyl-transferase (At5g17050), of which the latter one was more than 10-fold up regulated under Cold/Light (Table 2). The other genes encoding flavonoid biosynthesis proteins are located in the cytoplasm. Generally, these genes were more induced after Cold/Light than Cold/Dark treatment, with the exception of two flavonol synthase genes (At2g38240 and At5g05600). Additionally, there were two genes significantly up regulated only in Cold/Light conditions, cinnamoyl CoA reductase (At1g15950) and caffeoyl CoA 3-O methyltransferase (At4g34050) that are not related to flavonoid biosynthesis, but encode proteins for reconstruction of cell wall components like lignins, lignans, hydroxycinnamic acids, suberins, sporopollenins and cutins [36].

### Genes encoding proteins involved in carbon metabolism are not down regulated in Cold/Light or Cold/Dark treatments

Even though it is generally accepted that low temperature decreases carbon fixation (reductive carbon cycle) and inactivates Calvin cycle enzymes in chilling sensitive plants, this is not probably the case in chilling tolerant plants [37,38]. In accordance, we found no down regulation of Calvin cycle genes in Cold/Light or in Cold/Dark treatments. However, these transcripts were clearly down regulated after 8-hour dark treatment (see Additional file 4).

Genes encoding two sugar transporters, *ERD6*, (At1g08920) and a triosephosphate/phosphate translocator (At3g01550) were more up regulated under Cold/Light than Cold/Dark condition, and vice versa, two other sugar transporters, a sucrose/proton transporter (*SUC1*, At1g71880) and At4g36670 were up regulated only under Cold/Dark condition (Table 2). All these sugar transporters are membrane proteins with seven to twelve membrane spanning helixes, but do not have chloroplast targeting signals. The cytosolic fructose-bisphoshate aldolase gene (At4g26530) was slightly up regulated only after the Cold/Light treatment, whereas the corresponding plastidic fructose-bisphosphate aldolase gene (At4g38970) was up regulated upon both the Cold/Light and Cold/Dark treatments. In addition, there seems to be a differential expression between the genes involved in biosynthesis (starch synthase, At1g32900) and degradation of starch (α-amylase, At1g69830; β-amylase, At4g17090 and starch phosphorylase, At3g46970), of which the starch synthase and β-amylase were both up regulated more under Cold/Light than Cold/Dark condition, whereas the α-amylase and starch phosphorylase were down regulated under the Cold/Dark and Dark treatments.

Interestingly, we found three genes related to anaerobic carbon metabolism, which were clearly up regulated under Cold/Light condition, namely puruvate decarboxylase (*PDC1*, At4g33070), alcohol dehydrogenase (*ADH*, At1g77120) and L-lactate dehydrogenase (*LDH*, At4g17260) genes (Table 2).

### Differential expression of transcription factors

The results shown in Table 3 depict 48 transcription factors that were differentially expressed compared to control condition. Thirteen transcription factors were significantly up regulated only under Cold/Light condition, 17 both in Cold/Light and Cold/Dark condition, a few (6) preferably in Cold/Dark and 12 solely in the Dark condition. The largest group of differentially expressed transcription factors (14) belongs to various types of zinc finger family transcription factors.

**Table 3:** Genes encoding up regulated transcription factors that changed their expression upon different temperature and light treatments

AGI-code and Description	**Control**	**Cold/Light**	**Cold/Dark**	**Dark**
At2g23340 AP2 domain transcription factor, putative	0.9 ± 0.1	8.9 ± 1.1* ^(a)^	2.8 ± 0.9	1.0 ± 0.2
At5g63790 No apical meristem (NAM) protein, NAC-domain protein, (ANAC102)	1.2 ± 0.1	4.1 ± 0.4*	1.5 ± 0.6	0.9 ± 0.1
At2g47890 CONSTANS B-box like zinc finger family protein	1.1 ± 0.1	4.0 ± 0.5*	**1.6 ± 0.1**	1.0 ± 0.2
At4g08150 KNAT1 homeobox-related protein	1.0 ± 0.1	3.9 ± 0.5*	1.9 ± 0.6	0.7 ± 0.1
At5g05410 DRE binding protein (DREB2A)	1.0 ± 0.1	3.6 ± 0.3*	2.0 ± 0.5	0.9 ± 0.2
At5g04340 C2H2 zinc finger transcription factor – related	1.0 ± 0.1	3.3 ± 0.3*	**1.9 ± 0.1**	1.0 ± 0.1
At1g06040 zinc finger transcription factor STO	1.5 ± 0.2	3.3 ± 0.4*	1.7 ± 0.3	0.8 ± 0.2
At4g18390 TCP family transcription factor, teosinte branched1 protein	1.0 ± 0.1	3.0 ± 0.3*	**1.5 ± 0.1**	**0.6 ± 0.1**
At1g51700 Dof zinc finger protein ADOF1	1.0 ± 0.1	2.4 ± 0.5*	1.1 ± 0.2	0.8 ± 0.1
At4g34590 bZIP family transcription factor, ATB2/bZip11	1.1 ± 0.1	2.4 ± 0.6*	1.1 ± 0.1	0.9 ± 0.1
At5g54470 CONSTANS B-box zinc finger	0.9 ± 0.1	2.3 ± 0.2*	1.3 ± 0.3	1.0 ± 0.2
At5g44190 myb family transcription factor, (GLK2)	1.1 ± 0.1	2.2 ± 0.4*	1.1 ± 0.1	1.0 ± 0.3
At4g23750 AP2 domain transcription factor, (ERF)	0.9 ± 0.1	2.0 ± 0.3*	1.3 ± 0.1	2.0 ± 0.9
At4g25490 C-repeat/DRE binding factor 1 (CBF1) (DREB1B)	1.1 ± 0.1	**8.3 ± 2.7**	**4.0 ± 0.8**	1.0 ± 0.2
At1g27730 salt-tolerance zinc finger protein, C2H2-type, ZAT10	1.1 ± 0.2	**6.2 ± 3.7**	**7.2 ± 1.2**	1.1 ± 0.7
At5g57660 CONSTANS B-box like zinc finger family protein (COL5)	1.4 ± 0.2	**5.1 ± 1.0**	**4.1 ± 0.7**	**4.3 ± 0.5**
At2g46830 MYB-related transcription factor (CCA1)	1.0 ± 0.1	**3.8 ± 0.4**	3.7 ± 2.1	1.0 ± 0.2
At5g59820 zinc finger protein ZAT12	1.0 ± 0.1	**3.7 ± 1.1**	**2.3 ± 0.5**	1.1 ± 0.1
At1g49720 abscisic acid responsive elements-binding factor, ABF1/AtbZip35	1.0 ± 0.1	**3.4 ± 0.5**	2.1 ± 0.7	0.7 ± 0.1
At4g28140 AP2 domain transcription factor, RAP2.4	0.9 ± 0.1	**3.2 ± 0.6**	1.7 ± 1.3	1.0 ± 0.1
At1g13260 AP2 domain transcription factor, putative (RAV1)	1.1 ± 0.1	**3.1 ± 1.0**	**4.2 ± 0.8**	0.9 ± 0.1
At5g08790 No apical meristem (NAM) protein family, NAC-domain protein (ATAF2)	1.2 ± 0.2	**2.9 ± 0.5**	1.3 ± 0.6	1.0 ± 0.4
At5g37260 MYB family transcription factor	0.9 ± 0.1	**2.9 ± 0.4**	4.4 ± 2.2	1.2 ± 0.1
At4g12040 expressed protein zinc finger protein, AN1-like	1.0 ± 0.1	**2.8 ± 0.3**	**3.4 ± 0.5**	**2.2 ± 0.3**
At2g45820 remorin, a non-specific DNA binding protein	1.3 ± 0.2	**2.7 ± 0.5**	1.9 ± 0.6	**1.7 ± 0.2**
At3g52800 zinc finger – like protein zinc finger protein, AN1-like	1.0 ± 0.1	**2.6 ± 0.7**	**3.6 ± 0.8**	1.6 ± 0.5
At2g22430 homeobox-leucine zipper protein ATHB-6 (HD-Zip)	1.6 ± 0.2	**2.1 ± 0.5**	2.1 ± 0.5	1.6 ± 0.3
At5g02840 myb family transcription factor (SANT-domain)	1.2 ± 0.1	**2.1 ± 0.3**	1.7 ± 0.4	**2.0 ± 0.3**
At4g32800 AP2 domain transcription factor TINY	1.0 ± 0.2	**2.1 ± 0.1**	2.3 ± 0.8	**0.7 ± 0.1**
At5g52510 scarecrow-like transcription factor 8 (SCL8)	1.1 ± 0.1	**2.0 ± 0.2**	**3.6 ± 1.1**	1.1 ± 0.2
At3g55980 zinc finger transcription factor (PEI1), CCCH-type	0.9 ± 0.1	1.2 ± 0.7	3.8 ± 0.4*	1.1 ± 1.2
At3g07650 CONSTANS B-box like zinc finger (COL9)	1.0 ± 0.1	1.9 ± 0.3	3.5 ± 0.5*	1.5 ± 0.3
At2g21650 myb family transcription factor	1.0 ± 0.1	1.3 ± 0.1	**2.2 ± 1.5**	0.9 ± 0.2
At5g58900 myb family transcription factor (SANT Domain)	1.1 ± 0.1	**1.6 ± 0.2**	**2.2 ± 0.2**	0.9 ± 0.2
At2g03340 WRKY family transcription factor	1.1 ± 0.1	**1.7 ± 0.2**	2.1 ± 0.9	**0.4 ± 0.1**
At3g61260 DNA-binding protein-related DNA-binding protein (dbp)	1.1 ± 0.1	1.6 ± 0.4	**2.1 ± 0.5**	**5.2 ± 0.9**
At3g16770 AP2 domain transcription factor RAP2.3	1.4 ± 0.2	**1.7 ± 0.3**	**1.7 ± 0.3**	**8.5 ± 4.1**
At2g25900 CCCH-type zinc finger	1.3 ± 0.1	0.8 ± 0.1	1.1 ± 0.3	**5.1 ± 1.0**
At5g07100 WRKY family transcription factor SPF1	1.2 ± 0.1	1.0 ± 0.1	1.7 ± 0.5	**3.6 ± 0.5**
At1g02340 bHLH protein (HFR1)	1.3 ± 0.2	**1.6 ± 0.2**	**1.4 ± 0.1**	**2.8 ± 0.7**
At1g34370 zinc finger protein-related similar, C2H2-type	1.0 ± 0.1	1.2 ± 0.2	0.8 ± 0.1	**2.6 ± 0.2**
At2g42280 bHLH protein family	1.0 ± 0.1	1.0 ± 0.1	1.1 ± 0.1	**2.5 ± 0.1**
At3g59060 bHLH protein family	1.2 ± 0.1	0.7 ± 0.2	1.1 ± 0.2	**2.3 ± 0.3**
At5g56140 KH domain protein	1.1 ± 0.1	1.1 ± 0.1	1.1 ± 0.1	**2.3 ± 0.2**
At5g11260 bZIP protein HY5 identical to HY5	0.8 ± 0.1	**1.5 ± 0.2**	1.3 ± 0.1	**2.1 ± 0.2**
At4g17460 homeobox-leucine zipper protein HAT1 (HD-Zip protein 1)	1.2 ± 0.1	0.8 ± 0.1	0.9 ± 0.1	**2.1 ± 0.3**
At1g13450 DNA binding protein GT-1-related	1.1 ± 0.1	0.7 ± 0.1	0.9 ± 0.1	**2.1 ± 0.1**
At5g37720 RNA and export factor binding protein, putative transcriptional coactivator ALY, Mus musculus	1.2 ± 0.1	1.2 ± 0.1	1.0 ± 0.1	**2.0 ± 0.1**

**Control **represents internal variation (technical and/or biological) of different leaf samples from growth condition. Value ± s.e. indicates expression ratio of Treatment/Control after normalization ± standard error of the mean (n = 3–4). Genes in the table are listed according to decreasing expression ratios in different condition. The big groups of transcription factors that are up regulated at a given condition are underlined. ^(a)^Genes that differ significantly (Students t test p-value less than 0.05) in their expression between the **Cold/Light **and **Cold/Dark **condition is marked with an asterisk (*). Values **in bold **indicate the quality control of gene expression (a statistical test of differential expression for a specific condition, Students t test p-value less than 0.05).

Some of these transcription factors have been shown to be involved in oxidative stress, like ZAT12 At5g59820 [39] or in salt stress, like STO (At1g06040) and STZ/ZAT10 (At1g27730 [40]). The second largest group consisted of AP2-domain transcription factors (8), including two DRE binding proteins DREB1B (CBF1, At4g25490) and DREB2A (At5g05410) that have been well characterized in regulation of cold responsive and dehydration responsive genes, respectively [7,41]. Of these two genes, DREB2A (At5g05410) was significantly more induced by the Cold/Light than Cold/Dark treatment (Table 3, Figure 4), even though the low temperature is the main regulator of these transcripts [42]. In addition, a cold-responsive AP2-transcription factor RAV1 (At1g13260) was induced upon the cold treatment both in light and in darkness [43]. Other types of transcription factor genes were also found differentially expressed compared to control conditions, like 6 members of the *MYB *family of transcription factors, 3 members of homeobox related transcription factors, 3 members of the bZip transcription factors like *AtbZip35 *(*ABRE/ABF1*, At1g49720) and two genes encoding bZip family of chloroplast targeted transcription factors (*ATB2/AtbZIP11*, At4g34590 and *Hy5*, At5g11260), 2 members of the *WRKY *family of transcription factors, 3 members of the *bHLH *family of transcription factors, two *NAC*-domain family members of transcription factors and 11 other genes of DNA binding families of transcription factors. The expression of some transcription factor genes, three members of the AP2 and one member of the *MYB *family of transcription factors (*CCA1*, At2g46830) was verified using northern blot analysis (Figure 4). Despite the low expression of transcription factors in general, we found a good correlation between the northern blot and the microarray results (Table 3).

Since we were studying the effect of light in the cold acclimation process, it was of interest to find out whether the genes involved in circadian rhythm and/or phytochrome/cryptochrome related light sensing processes were likewise affected. However, we did not find any differences between the Cold/Light and Cold/Dark treatments in the transcriptional expression of genes encoding phytochrome/cryptochrome related transcription factors, light regulators or light receptors (Data not shown). Instead, we found a clear differential expression of these photoreceptor-responsive genes after the Dark treatment.

### Evaluation of the correlation between the transcript and protein levels

**Figure 7:**
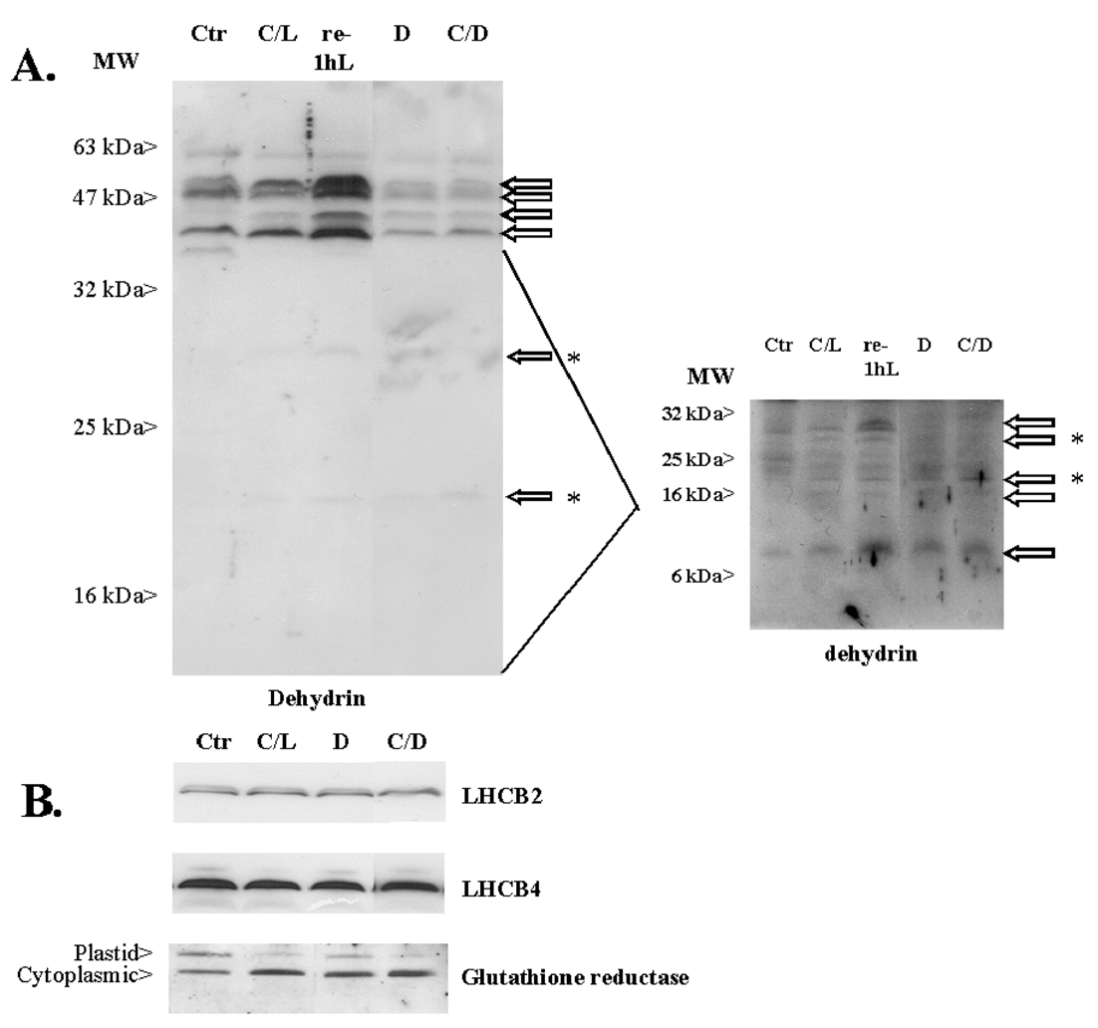
**Western blot analysis of dehydrin proteins (A) and of LHCB and glutathione reductase proteins (B)**. Protein samples isolated from *Arabidopsis *leaves under growth condition (Ctr), after the Cold/Light (C/L) treatment, subsequent recovery for one hour at normal growth conditions (re-1hL), after 8-hour Darkness (D) and after the Cold/Dark (C/D) treatment. A typical result is presented out of three independent western blot experiments.

Some genes that showed large expression changes at the transcript level were also analyzed at the protein level by western blotting (Figure 7). This analysis was limited to low temperature inducible dehydrins like XERO2 and ERD10 and to some photosynthesis related genes, which were strongly up regulated, especially under the Cold/Light condition. Figure 7A depicts the dehydrin proteins and their relative quantities under Control, Cold/Light, Cold/Dark and Dark conditions. Strong up regulation recorded at transcript level, both under Cold/Light and Cold/Dark did not occur at the protein level. On the contrary, under Cold/Dark condition the protein amounts were decreased. However, it is interesting to note that the amount of proteins did increase when the plants were allowed to recover for one hour at normal growth temperature (re-1hL). Similarly, despite strong up regulation of *LHCB *and glutathione reductase transcripts, the protein levels of chloroplast targeted LHCB proteins and glutathione reductase protein remained nearly unchanged during the Cold/Light and Cold/Dark treatments (Fig. 7B). Another glutathione reductase gene (At2g24170), which however, was not present in our cDNA array, encodes a cytoplasmic protein, and this protein showed increased amounts both under Cold/Light and Cold/Dark conditions. Based on these few protein analyses, it is clear that the transcript up regulation is not necessarily reflected in the increased protein contents; in fact the opposite might occur as in the case of dehydrin proteins in darkness. We are presently undertaking a proteome study, in order to specify how the transcript levels of highly responsive genes are related to respective proteins levels.

### Transcription factors were bound to corresponding response elements according to their expression level

**Figure 8:**
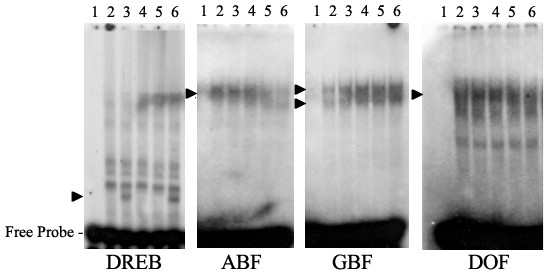
**Electrophoretic mobility shift assay (EMSA) indicating DNA-protein interactions under different light and temperature conditions**. The oligonucleotide DNA probes are shown below each EMSA experiments. *In vitro *translated protein extracts that were used in EMSAs are indicated by the numbers: 1 no protein extract, 2 Control (growth condition), 3 Cold/Light, 4 Cold/Light and subsequent recovery for one hour at normal growth condition, 5 Darkness and 6 Cold/Dark. Inducible DNA-protein interactions are indicated by arrow heads.

Electrophoretic mobility shift assay (EMSA) was used to demonstrate the interaction between the DNA binding proteins (i.e. putative transcription factors) and the corresponding response elements present in the promoter regions of low temperature/light responsive genes. For this purpose, the mRNA isolated from differently light and low temperature treated leaf rosettes was translated *in vitro *and the binding of proteins to four DNA response elements was tested (Figure 8). Since, the *in vitro *translation mixture contains a variety of different DNA binding proteins; it is possible that several transcription factors bind to the same response element. As demonstrated in Figure 8, the *in vitro *translated protein mixture originating from the Cold/Light or Cold/Dark samples contained specific binding activity to the DRE response element, thus most probably containing a low temperature induced DRE binding (DREB) protein. Interestingly, only one hour recovery at growth temperature after the Cold/Light treatment was enough to abolish this DNA-protein interaction (Figure 8), in accordance with a decrease of mRNA encoding the DREB proteins (Data not shown). The translated protein mixtures also contained proteins binding to ABA and DOF responsive elements but no increase in the binding activity to these elements was observed either by the Cold/Light or by the Cold/Light and subsequent 1h-recovery treatments of plants. However, less binding of transcription factors to these elements occurred when plants had been treated for 8-hours in Darkness or in the Cold/Dark condition. In contrast, increased binding of *in vitro *translated proteins to the GBF-element occurred under all treatments as compared to control.

## Discussion

### Light has a significant effect on the expression of transcription factors during exposure of plants to low temperature

The work presented here addresses for the first time the role of light in the cold acclimation process of *Arabidopsis *at the transcript level using microarray techniques. In this respect, the induction of transcription factors and their role in transcriptional activation of cold-responsive genes is highly important (Tables 3 and 4). Approximately 1700 transcription factors have been identified in *Arabidopsis thaliana *genome of which only a small fraction is genetically characterized [44].

**Table 4:** Summary of Transcriptional Regulation of Target Genes (Table 2) and Transcription Factor Genes (Table 3). The Table Represents the Number of Response Elements Present in the Promoter Region of Differently Expressed Cold/Light (C/L) or Cold/Dark (C/D) or Dark (D)-Responsive Genes. Promoter Analysis Was Performed within 500 bps Upstream of Transcription Start Site using a Genomatix™ and Athena Programs. Response Elements that Are More than 90% Same within a Group of Expressed Genes are underlined According to Genomatix Gene2Promoter Analysis. Enriched Response Elements within the Same Groups of Genes (P-value < 10e^-3^) Are Indicated as Underlined and Bold According to Athena Visualization Program [71].

Response elements and conditions under which the elements induce genes	DREB^1^	ABRE^2^	AHBP^3^	GBOX^4^	GTBOX^5^	LREM^6^	CCAF^7^	HSF^8^	CGCG^9^	SALT^10^	DOFF^11^
	
	Cold	ABA	ABA	ABA/Light	Light	Light	Circadian	Heat	Calcium	Salt	Carbon
**Target genes, AGI Code, description**											

At1g56600, Galactinol synthetase.C/L	**2**	**7**	4	7	6	2	2	0	1	0	1
At1g09350, Galactinol synthase. C/L, C/D	**1**	**3**	1	5	2	1	0	0	0	0	1
At3g50970, XERO2, C/L, C/D	**3**	**0**	5	0	1	3	0	0	0	0	4
At5g52310, RD29A, C/L, C/D	**4**	**1**	2	3	4	0	0	0	2	1	3
At1g20450, ERD10, C/L, C/D	**3**	**2**	3	0	3	2	0	1	1	1	3
At5g15960, KIN1, C/L	**1**	**4**	0	4	5	2	0	1	0	0	1
At5g66400, RAB18, C/L	**1**	**3**	4	2	5	0	0	0	2	1	2
At1g20340, PC, C/L	**1**	**2**	1	5	5	4	0	1	2	3	4
At4g11600, GPX, C/L	**1**	**3**	1	8	6	2	1	0	0	1	1
At1g77120, ADH, C/L	0	**3**	2	3	6	1	**1**	1	0	0	4
At3g08940, LHCB4.2, C/L	0	**2**	2	3	2	3	**0**	0	0	1	1
At3g27690, LHCB2.4, C/L	0	**1**	6	6	1	2	**3**	1	0	0	1
At2g05070, LHCB2.2, C/L	0	**2**	3	3	2	1	**1**	0	0	1	2
At1g29930, LCHB1.3, C/L	0	**3**	1	5	3	7	**4**	0	0	0	1
At5g67030, ZEP, C/L	0	**3**	1	6	3	0	**4**	0	0	0	1
At5g13930, Chalcone synthase. C/L	0	**4**	1	3	1	3	**0**	1	0	0	2
**Transcription factor genes**											
At4g25490, DREB1B, C/L, C/D	0	**3**	2	5	5	1	0	0	0	0	2
At5g05410, DREB2A, C/L, C/D	0	**1**	3	2	3	5	1	2	0	0	1
At2g46830, CCA1, C/L, C/D	0	**3**	3	2	4	1	0	1	2	0	3
At1g49720, ABF1, C/L, C/D	0	**0**	0	0	2	2	1	2	0	0	5
At1g13260, RAV1^13^, C/D, C/L	0	**1**	3	0	7	1	0	2	0	0	2
At1g51700, ADOF1, C/L	0	**0**	0	0	9	1	3	1	0	1	2
At4g34590, ATB2-GBF6, C/L	0	**0**	11	0	6	2	5	0	0	0	4
At5g63790, ANAC102^12^, C/L	0	**6**	1	10	1	0	0	0	0	0	2
At5g08790, ATAF2^12^, C/L	1	**1**	3	6	3	2	1	0	0	0	3
At2g42280, MYC ^14 ^D	0	**2**	2	0	5	3	1	0	0	0	2
**Miscellaneous, Dark induced genes**											
At3g12580, HSP70, C/D	1	0	3	0	6	1	2	5	1	0	2
At2g32950, COP1 regulatory protein, D	1	0	3	3	5	0	0	1	2	0	1
At4g02440, F-box protein, ZGT, D	0	0	0	1	3	0	0	0	2	0	2
Control set of random genes, no changed expression in C/L, C/D or D condition *											
At2g37460, Root nodulin MtN21	0	0	0	0	4	0	1	0	0	0	0
At4g12550, Seed lipid transfer protein LTP	0	0	3	0	6	0	1	1	0	0	0
At3g56020, 60S ribosomal protein L41	0	0	0	0	0	0	2	0	3	0	1
At4g31700, 40S ribosomal protein RPS6	0	0	5	1	1	0	1	0	0	0	1
At3g47600, MYB94	0	0	1	1	1	0	0	0	0	1	1
At1g73460, protein kinase	0	0	0	0	1	0	2	1	2	0	1

Footnotes 1–14: References for response elements found in *Arabidopsis thaliana*.1) [13]; [72] 2) [12] 3) [73]; [74] 4) [75]; [76] 5) [77]; [78] 6) [79]. 7) [80]; [81] 8) [82]; [83] 9) [84] 10) [85]; [86] 11) [87] 12) [88] 13) [89] 14) [90]

* Altogether, the promoter regions of 1000 genes in the Control group were tested with similar results.

Previous studies have demonstrated that cold acclimation involves a rapid up regulation of genes encoding CBF transcriptional activators [7] and other ERF/AP2 domain proteins, known also as DRE binding (DREB) proteins [45]. It is of interest that two genes encoding AP2 domain transcription factors (DREBA2 (At5g05410) and a previously uncharacterized *AP2*-domain transcription factor (At2g23340) were all significantly more up regulated in Cold/Light than in Cold/Dark (Table 3), being in line with similar enhanced up regulation of the well-established cold responsive genes (Table 2, Figure 4). Our transcript profiling together with promoter analysis (Table 4, Additional file 5 and Figure 8) suggest a concerted effect of low temperature and light in the up regulation of several cold responsive genes.

We also identified a number of transcription factor genes specifically induced only under the Cold/Light condition, although the light intensity was not changed upon a shift of plants to low temperature. Therefore we anticipate that signals from chloroplasts, modified by low temperature [19], are important in regulation of these genes. They include genes encoding two NAC domain proteins (*ANAC102*, At5g63790 and ATAF2, At5g08790), three AP2/ERF-domain proteins (At2g23340, At5g05410 and At4g23750), five zinc finger proteins (At2g47890, At5g04340, At1g06040, At1g51700 and At5g54470), a homeobox (At4g08150), a TCP family (At4g18390), a bZIP family (At4g34590) and a myb family (At5g44190) of transcription factors. Only three of these genes (At5g63790, At4g28140 and At4g23750) seem not to be subject to regulation by various light treatments as revealed by using the GENEVESTIGATOR program, [10]. It is conceivable that these transcription factor genes respond to changes in the function of the photosynthetic machinery and related changes in chloroplast redox conditions and/or the production of different metabolites derived from CO_2 _fixation [4]. These transcription factors are, in turn, likely to induce the Cold/Light specific nuclear encoded transcripts (Tables 2 and 3), many of which are characterized by a chloroplast targeting signal and are involved in different protective functions, including the biosynthesis of carotenoids, reactive oxygen scavenging enzymes and components of the phenylpropanoid pathway as well as in the production of ABA.

### Environmental conditions modulate the expression of transcription factor genes and their target genes via the same response elements

An important fact was discovered when examining the response elements in the proximal promoter regions of both the transcription factor genes and their target genes (Table 4, Additional file 5). Similar response elements in the promoter regions of these both gene groups were evident. Environmental conditions thus seem to regulate via signalling/metabolic cascades the expression of both the transcription factor genes and their target genes. To some extent this was expected since the microarray data would produce information of genes having a similar expression pattern under similar stress conditions. In Table 4 we have selected and analysed the promoter elements of some most up regulated genes either under Cold/Light or under both the Cold/Light and Cold/Dark conditions. It is clear that the increasing number of DRE binding elements present in the most highly expressed Cold/Dehydration responsive genes (*XERO2*, *RD29A *and *ERD10*) is related to their transcriptional efficiency independently of light (Table 2, Figure 8). These elements are also expected to play an important role in light-dependent regulation of photosynthesis-related as well as other light-regulated genes.

It is conceivable that the light and/or ABA-responsive elements are additionally involved in transcriptional regulation of many cold responsive transcription factor genes, since they do not contain any DRE binding elements (see Table 3). Indeed, it was interesting to note the absence of positive feedback loops for transcriptional regulation of transcription factor genes, as they did not appear to have their own response elements in the promoter regions (except *ADOF1*). The promoter region of different genes depicted in Table 4 contains also a number of other response elements capable of transcriptional regulation according to circadian, heat, calcium, salt or carbon status of the cells. Thus, it appears that environmental cues have a direct effect on the transcriptional network via signalling events, which are driven by cellular and metabolic processes. Transcription factor genes responding to these signals, concomitantly with their target genes, then have a capacity to specifically amplify the expression of these target genes.

### Putative light signalling pathways involved in low temperature regulation of gene expression

The obvious question is how light affects the low temperature signalling cascades involved in induction of transcription factors and their target genes. Earlier studies have pointed out the necessity of light for induction of genes through the C-repeat/dehydration responsive elements in response to low temperature, being mediated by phytochrome B [46]. This mechanism is likely involved in the case of DREB/CBF and related transcription factors [43], whose expression was clearly enhanced by Cold/Light as compared to Cold/Dark condition.

A MYB-related transcription factor CCA1 (At2g46830) is another well-characterized phytochrome B linked factor that has previously been described to be involved in light-regulation of genes encoding chloroplast targeted proteins like the light harvesting (LHCII), ELIP1 and chlorophyll biosynthesis (e.g. HEMA1) proteins [47]. Our results, however, demonstrate that both Cold/Light and Cold/Dark conditions induce the *CCA1 *gene, whereas the accumulation of several *LHCB *and *ELIP1 *transcripts occurs only upon the Cold/Light treatment (Tables 3 and 4). It is therefore possible that CCA1 protein is post-transcriptionally modified only in Cold/Light by a CKII kinase induced phosphorylation, which has been shown to stimulate the binding activity of CCA1 to the target DNA regulatory regions [48,49].

Our results indicated that ABA biosynthesis genes are specifically induced under Cold/Light treatment. A clear indication of this was the induction of the first gene in ABA biosynthesis, the zeaxanthin epoxidase (*ZEP*) gene [31,33]. In previous microarray experiments using ABA-treated plants (100 μM), 22 genes encoding transcription factors were induced [6], many of which behaved similarly in our Cold/Light experiments. Our study and the studies above suggest that ABA may act as a chemical signal via induction of bZip (ABRE, ABF, G-box), MYC (bHLH), homeodomain-leucine zipper (HD-Zip) and MYB transcription factors (Table 4, Figure 8 and Additional file 5). Moreover, an increased level of ABA has been shown to increase for example the expression of *LHCB *genes in microarray experiments, similarly to our Cold/Light treatment [5,6]. ABA-marker genes (listed in supplemental material of Nemhouser et al. [50]) were also analyzed against our gene lists of two-fold up and down regulated genes in the Cold/Light and Cold/Dark conditions. Out of 119 ABA marker genes present in our array, the up regulation of twelve genes was found to be specific to the Cold/Light condition, up regulation of nine genes was common for both conditions and only two were specific to the Cold/Dark condition (see also Additional File 6). The initial steps of ABA biosynthesis are occurring in the chloroplasts; only the last three steps are cytosolic [31,33]. Thus, it is possible that ABA may have a light dependent role in inducing transcription via ABA-responsive transcription factors, but in darkness also decreasing the transcription of genes encoding ABA-responsive transcription factors (Figure 8). Interestingly, the promoter analysis in Table 4 indicates that almost all genes induced only by Cold/Light contained the ABRE, AHBP (HD-Zip) and G-box DNA binding motifs. This result is further supported by the fact that a significant over-presentation of these promoter motifs was found in those genes that were clearly up regulated only under Cold/Light or under both Cold/Light and Cold/Dark conditions but not in those genes up regulated only in Cold/Dark condition (Additional file 5). The exact mechanism by which ABA/Light induces the up regulation of these transcription factor genes remains to be elucidated in more detail.

We also considered the reduction state of thylakoid electron carriers and the production of H_2_O_2 _in chloroplasts as Cold/Light-induced stress signalling pathways [51]. Both physical characterization of *Arabidopsis *plants [Figure 6, [22]] and our microarray data, indicated that production of some ROS species in the chloroplasts was induced under the Cold/Light condition and some genes encoding ROS scavenging proteins were turned on (Table 2). We found a specific up regulation only under Cold/Light treatment of three genes encoding chloroplast ROS scavenging proteins, an iron superoxide dismutase (*FeSOD*) and two glutathione dependent peroxidases (*GPX*) [52] (Table 2). However, there seems to be a specificity of ROS signalling in the Cold/Light treatment. Indeed, no induction of glutathione-ascorbate cycle genes (Table 2), like ascorbate peroxidases, monodehydroascorbate or dehydroascorbate peroxidases known to become induced under excess light stress [11,53-56] was recorded upon the Cold/Light treatment. Thus, it is evident that our Cold/Light treatment did not induce such a high PSII excitation pressure as was reported in earlier experiments by Huner and coworkers [19], where a high light intensity treatment or a low temperature treatment in combination with moderate light intensity was applied. Even though our Cold/Light treatment induced detectable levels of ROS species, probably originating from chloroplasts, it is possible that the plants exposed Cold/Light were able to scavenge most of these oxygen radicals. Our previous microarray experiments with *Arabidopsis *[4] excluded the role of PSII excitation pressure in regulation of gene expression up to the light intensity of 450 μmol photons m^-2 ^s^-1^. Such moderate high light condition induced a gene expression pattern very similar to the Cold/Light condition, particularly in respect to the photosynthesis and oxidative stress related genes (See Supplemental Material in Piippo et al., 2006 [4], and Table 2). Apart many similarities between Cold/Light and moderate high light induced genes, the major differences were detected in the expression of *LHCB *genes. Under moderate high light these genes were mainly down regulated except for the gene encoding ELIP1, whereas under Cold/Light condition these *LHCB *genes were clearly up regulated.

The Cold/Light condition induced reduction of both the PQ pool and also of the stromal electron acceptors on the reducing side of PSI, as can be deduced from the phosphothreonine blots (Figure 1, [23]). It has frequently been suggested that several nuclear genes respond to the redox state of the PQ pool to regulate photosystem stoichiometry and light harvesting capacity as well as antioxidant scavenging systems [57-60]. Our results however, are in variance with this hypothesis as evidenced by strong up regulation of *LHCB *genes under Cold/Light conditions that induce a reduction of the PQ pool.

Sugar metabolism/signalling is yet another potential source for regulation of nuclear genes [4,15,61-63] and is likely to cause drastic changes in gene expression between Cold/Light, Cold/Dark and Dark treatments. A shift from photosynthesis to respiratory sugar metabolism is a probable cause for the changes detected in the gene expression. For example, Dark treatment under normal growth temperature induced twice as many genes as the Cold/Dark treatment (Figure 2B, Additional files 7 and 8). Starch synthesis is evident under Cold/Light condition (Table 2), whereas soluble sugars are expected to increase under Cold/Dark and especially under Dark condition. The effect of signalling sugar molecules on nuclear gene expression might be exerted via DOFF or WRKY (SPF1) elements present in both the transcription factor genes and their target genes (Table 3).

## Conclusion

Acquisition of cold hardiness and freezing tolerance in *Arabidopsis *plants is enhanced by a low temperature treatment, particularly when performed in light as compared to darkness. Here we show that light is indeed required for low temperature induced transcriptional expression of several transcription factors and genes involved in metabolic pathways, which are essential for development of cold hardiness. It is likely that several light-signalling pathways, originating from chloroplasts by production of redox active molecules and photosynthesis end products are cross talking with various pure low temperature induced pathways, which in turn probably rely on changes in Ca^2+ ^efflux across cell membranes [64], thereby together resulting in the maximation of cold acclimation [65]. Light was shown to enhance the expression of well-known cold-responsive genes (Table 2), which is in line with presence of multiple ABRE and DOF response elements in the promoter regions of several cold-induced genes (Tables 3 and 4). Besides enhancing the expression of cold-responsive transcription factors and their target genes, Cold/Light also had unique effects. Most distinctively, the combination of Cold and Light was particularly needed for up regulation of several genes encoding enzymes involved in biosynthesis of hormones (ABA) and various other compounds essential for scavenging of reactive oxygen species, for protection of membranes and for remodulation of the components involved in electron transfer reactions in the thylakoid membrane in order to optimize their function at low temperature.

## Methods

### Plant material and growth conditions

*Arabidopsis thaliana *ecotype Col-0 seeds were germinated in 50% vermiculite/50% soil and grown in controlled-environment chambers at 23°C and under 100 μmol photons m^-2 ^s^-1 ^(8-hour-photoperiod) for 8 weeks. A short day photoperiod was used to prevent bolting (flower shoot formation) and thus keep the plants under vegetative growth. The relative humidity was controlled during growth and low temperature treatments and kept constantly at 60%. Cold treatments for 8 hours were performed at 3°C (measured from the bottom of the leaf using digital thermocouple). To allow full CO_2 _assimilation before the treatments, plants were transferred after 2 hours of the beginning of light period directly from growth temperature to the low temperature treatment. The cold treatments were performed either in the light (100 μmol photons m^-2 ^s^-1^) or in the dark. The dark treatment at 23°C for 8 hours was also performed after 2 hours of the beginning of light period. Controls for Cold/Light, Cold/Dark and Dark samples were treated at 23°C in the light (100 μmol photons m^-2 ^s^-1^) the same 8 hours. Internal variation in results was controlled by Blanc hybridizations of leaf material collected from growth conditions (designated as Control in Tables 2 and 3). After treatments, 2 to 3 g of leaf material from 4–6 individual plants was collected and directly frozen in liquid nitrogen.

### PSII activity measurements

Fluorescence measurements of *Arabidopsis *leaves after different light and temperature treatments were performed using a PAM2000 fluorometer (Walz, Germany). Detached leaves were kept in the dark for 30 minutes prior to the detection of variable (Fv) and maximal (Fm) fluorescence. Steady-state rates of oxygen evolution were measured using Hansatech DW1 O_2 _electrode and saturating light intensity in the presence of 1 mM 2,5-dimethyl-*p*-benzoquinone (DMBQ) as an artificial electron acceptor and at Chl concentration of 10 μg Chl mL^-1^. Thylakoid membranes were isolated according to [66]. The chlorophyll content was determined according to [67].

### Western blot analysis

After specific treatments of plants, the leaves were detached and immediately frozen in liquid nitrogen. Total proteins were isolated simultaneously with RNA isolation using TriZol reagent (Invitrogen Inc., USA) according to manufacture's instructions. Protein samples (25 μg of total protein/well) were solubilized in 6 M urea Laemmli buffer and run in a 6 M urea SDS-PAGE. Proteins were detected using dehydrin antibody (Stressgen Bioreagents, Canada), specific LHCB2 and LHCB4 antibodies (Agrisera, Sweden) and glutathione reductase antibody (a gift from Helen Reynolds/Philip Mullineaux, John Innes Institute Norwich, UK).

All buffers for thylakoid membrane isolation contained 10 mM NaF. Thylakoid samples corresponding to 1 μg Chl per lane were run in SDS-PAGE, and thylakoid phosphoproteins were immunodetected with phosphothreonine antibody (New England Biolabs) using chemiluminescence for detection (ECL, Amersham biosciences, UK).

### 77 K fluorescence

77 K fluorescence emission spectra of thylakoid membranes were measured with a diode array spectrophotometer (S2000, Ocean Optics, Dunedin, Florida, USA) equipped with a reflectance probe. Fluorescence was excited with light below 500 nm (defined with LS500S and LS700S filters, Corion Corp., Holliston, MA) and the emission was recorded between 600 and 800 nm.

### Detection of oxidative stress by 3,3'-diaminobenzidine (DAB) uptake method

Oxidative stress was detected by DAB method [68]. Leaf samples from the Control, Cold/Light and Cold/Dark were placed under vacuum in a solution containing 1% DAB (w/v) in 10 mM Mes (pH 5.8) for 1 h. The leaves were cleared by boiling in ethanol (96%) for 5 min. The products of oxidative stress were visualized as a reddish-brown coloration.

### RNA isolation and cDNA labelling

Total RNA was first isolated using TriZol-reagent (Invitrogen, USA) according to manufacturer's recommendations and used for further isolation of mRNA by Dynabeads mRNA Purification Kit (Dynal, Biotech, Norway). 250 μg of total RNA yielded 2.5–5 μg of mRNA. For Cy3 (control) and Cy5 (treated) cDNA labelling (vica versa in dye swap), 1 μg of poly(A) mRNA was labelled with (dT)_12–18 _primers (Amersham Biosciences, UK) with direct incorporation of either Cy3 or Cy5 dUTP using Superscript II Reverse Transcriptase (Invitrogen, USA). RNA was degraded and the labelled cDNA products were further purified using Microcon ^® ^YM30 columns (Millipore, USA). The quantity of labelled cDNAs was measured using Nanodrop ND-1000 spectrophotometer (Nanodrop Technology's, USA) and the quality was checked on 1% agarose gel prepared in TAE (Tris/Acetate/EDTA) buffer.

### Microarray hybridizations and scanning

Cold/Light, Cold/Dark and Dark samples were hybridized against samples kept the same 8-hours in normal growth conditions. Additionally, control hybridization was made against leaves taken from different individual *Arabidopsis *plants after 10 hours of the beginning of the light period. *Arabidopsis *cDNA microarray slides are based on the GEM1 clone set (8000 ESTs) purchased from InCyte Genomics, Palo Alto, CA, USA [4]. The 8 k cDNA array was spotted as triplicate on the slides allowing three technical replicates upon each hybridization. Slides were UV-cross linked (90 mJ/cm^-2^) and prehybridized with 1% BSA (fraction V) in 5 × SSC, 0.1% SDS for 30 min at 50°C and washed with 2 × SSC and 0.2 × SSC for 3 min. After centrifugation (500 × g for 10 sec) the slides were used for the hybridizations during the same day. The labelled cDNAs were combined (10 to 20 pmol of labelled Cy3 and Cy5 cDNAs) in the total volume of 80 μl (3 × SSC, 0.65× Denhardt's and 0.3% SDS). Hybridization was performed in a sealed chamber (Corning, USA) overnight at 65°C. The arrays were washed at room temperature with 0.5 × SSC/0.1% SDS for 15 min, twice with 0.5 × SSC/0.01% SDS for 5 min and twice with 0.06 × SSC for 1 min and spinned dry for 10 sec with microarray centrifuge. The slides were scanned using ScanArray Express 5000 device (GSI Lumonics, USA) and the spot intensities were quantified using software provided by the same device (Scan Array Express Microarray Analysis System 2.0, Perkin-Elmer, USA). Visually bad spots or areas on the array and low intensity spots were excluded.

### Microarray data analysis

The spot intensity data was transferred to GeneSpring GX 7.3 software (Agilent Technologies, USA). From the three technical replicates of each slide, the median intensity value was taken for further calculation of intensity dependent normalization of spots for each slide (Lowess, Per Spot and Per Chip). Biological experiments consisted of three to four independent biological replicates (each biological replicate starting from a new set *Arabidopsis *seeds to grow a new set of plants). Control and Cold/Light treatments consisted of four biological replicates, whereas Cold/Dark and Dark treatments consisted of three biological replicates. The correlation co-efficient between the biological replicates varied from 0.6 to 0.8, which was considered relevant for this kind of analysis. Of Lowess normalized data, GeneSpring program calculated the ratios of up or down regulation of genes. Next the normalized values of up or down regulated genes were tested for statistical confidence. The induction or repression of a gene should be statistically significantly different from a ratio of 1.0 determined with Students t test in GeneSpring (termed as quality control). After Benjamini and Hochberg false discovery rate (FDR) correction for multiple testing, a false discovery rate of 0.05 or less was considered statistically significant. In addition, standard error of a mean (s.e., n = 3 or 4) was calculated for each normalized ratio value presented in the Tables. All original data containing normalized ratios of means, standard errors of the mean and t-test p-values (quality control), can be found in the Additional files 2 to 4 and 7 to 10. To find out whether the difference in gene expression between Cold/Light and Cold/Dark treatments was statistically significant, a Students t test was performed. The risk level set was at 0.05 (p < 0.05, marked as * in Tables 2 and 3). *Arabidopsis *genes encoding proteins potentially targeted to different cellular compartments, like chloroplast, mitochondria or secretory pathway were searched in GeneSpring using TargetP based MIPS database [69]. Our microarray contained 1260, 649, and 1133 genes potentially targeted to chloroplast, mitochondria and the secretory pathway, respectively. All the raw data has been submitted and accepted to ArrayExpress (EBI) database in line with the MIAME principles of publishing microarray data (submission accession number: E-MEXP-1068).

### Northern blot analysis

RNA was isolated from *Arabidopsis thaliana *leaves (2–3 g) using the TriZol-reagent method according to the manufacturer's (Invitrogen, USA) instructions. Total RNA samples (10 μg per lane) were denaturized using glyoxal at 50°C for 1 hour, separated on a 1.2% agarose gel and blotted onto nylon filters. Blots were hybridized overnight at 65°C with probes prepared from cDNA fragments that were previously used for spotting of the cDNA slides. Radioactive labelling of the probes with ^32^P-dCTP was performed using a Prime-a-Gene Kit (Promega, USA). Blots were washed finally at 50°C, 1 × SSC, 0.1% SDS and visualized by autoradiography. The mRNA expression levels were analyzed and quantified using a computerized image analysis scanner (ChemiImager™ 8000, Alpha Innotech Corp, USA).

### Electrophoretic mobility shift assay (EMSA)

Electrophoretic mobility shift assay was performed according to Kvietikova et al., 1995 [70]. Instead of using nuclear extracts, we used proteins translated *in vitro *from the same mRNA used for the microarray experiments. *In vitro *translation was performed according to Wheat Germ Extract Technical manual (L4380) (Promega, USA). Translation efficiency and quality of *in vitro *translation was checked using ^35^S L-methionine for labeling of the newly synthesized proteins and the products were visualized by 2D-gel electrophoresis and autoradiography (results not shown). For production of non-radioactive *in vitro *translated proteins, 1 mM L-methionine was used. Five double stranded oligonucleotide probes were made: DREB 5'tgactaCCGAcatgagttcc3', ABF 5'ccttgtccacGTGTatcatc3', DOF 5'atcttatatAAAGcaccatt3', and GBF 5'cttgtccACGTGtatcatca3'. These double stranded oligonucleotide probes were end-labeled with 32P-γ-ATP using T4 Polynucleotide Kinase (Fermentas, Litauen). DNA-protein binding reactions were performed at 10°C for 30 min in a total volume of 20 μl containing *in vitro *translation mix (100 μg protein), including the newly translated proteins, 0.5 × 10^4 ^cpm oligonucleotide probe, 100 ng poly (dI-dC) (Amersham Biosciences, USA) as non-specific competitor in 10 mM Tris/HCl pH 7.5, 50 mM KCl, 50 mM NaCl, 1 mM MgCl_2 _1 mM EDTA, 5 mM DTT, and 5% glycerol. DNA-protein samples were separated in 5% non-denaturing polyacrylamide gels for 2 hours at 150 V using TBE as a buffer. Gels were then dried and exposed to autoradiography at -80°C for 48 hours.

## Authors' contributions

AJS carried out microarray experiments from plant material to data analysis; Fv/Fm measurements, Western and Northern blot analysis, EMSAs and writing the article. MP participated in technical support. YA carried out the oxidative stress measurements in leaves, phosphothreonine immunoblots, 77 K and oxygen evolution measurements. NB participated in creating the microarray slides. E-M A participated in planning the experiments, reading and revising the manuscript. All authors read and approved the final manuscript.

## Supplementary Material

Additional file 1 Clustering of all genes under three different conditions (three biological replicates are shown for Cold/Light and Cold/Dark treatments and two for the Dark treatment). The brightness of colours indicates the expression level of the genes; magenta indicates up regulation and green down regulation of clustered genes. Click here for file

Additional file 2 Up or Down Regulation of **Cold/Light and Cold/Dark **Responsive Genes at Least Two-Fold (p-value less than 0.05 at least in condition) Click here for file

Additional file 3 Up or Down Regulation of **Cold/Light-Responsive Genes **at Least Two-Fold (p-value less than 0.05 at least in one condition) Click here for file

Additional file 4 Down Regulation of Cold/Light, Cold/Dark and Dark Responsive genes Containing a Chloroplast Targeting Signal (p-value less than 0.05 at least in one condition) Click here for file

Additional file 5 Enriched transcription factor binding sites (TFs) were checked in three different groups of genes that were up regulated more than two fold: Only Cold/Light, Both Cold/Light and Cold/Dark and Only Cold/Dark conditions. Genes for the analysis were taken from Additional files 2, 3 and 8, respectively. Significant over-presentation of promoter motifs within the group (P-value < 10e^-4^) is indicated by ***. Analysis was performed using Athena visualization tool [71]. Click here for file

Additional file 6 Visualising gene expression only under Cold/Light, Cold/Dark, Dark, conditions using MAPMAN analysis [91]. Following MAPMAN schemes were used: Metabolic Overview, Regulation Overview and Cellular Function Overview. Up regulated genes are shown in blue and down regulated genes in red. Visualized data is based on Additional files 3, 7 and 8. Click here for file

Additional file 7 Up or Down Regulation of **Dark-Responsive Genes **at Least Two-Fold (p-value less than 0.05 at least in one condition) Click here for file

Additional file 8 Up or Down Regulation of **Cold/Dark-Responsive Genes **at Least Two-Fold (p-value less than 0.05 at least in one condition) Click here for file

Additional file 9 Up or Down Regulation of **Cold/Dark and 8 h Dark-Responsive Genes **at Least Two-Fold (p-value less than 0.05 at least in condition) Click here for file

Additional file 10 Up Regulation of Cold/Light, Cold/Dark and Dark-Responsive Genes Containing a Chloroplast targeting Signal (p-value less than 0.05 at least in one condition) Click here for file
