# Supplementary figures and images for: Light has a specific role in modulating Arabidopsis gene expression at low temperature
Source: BMC Plant Biol. 2008 Jan 29;8:13. doi: 10.1186/1471-2229-8-13 (PMC2253524; doi:10.1186/1471-2229-8-13)

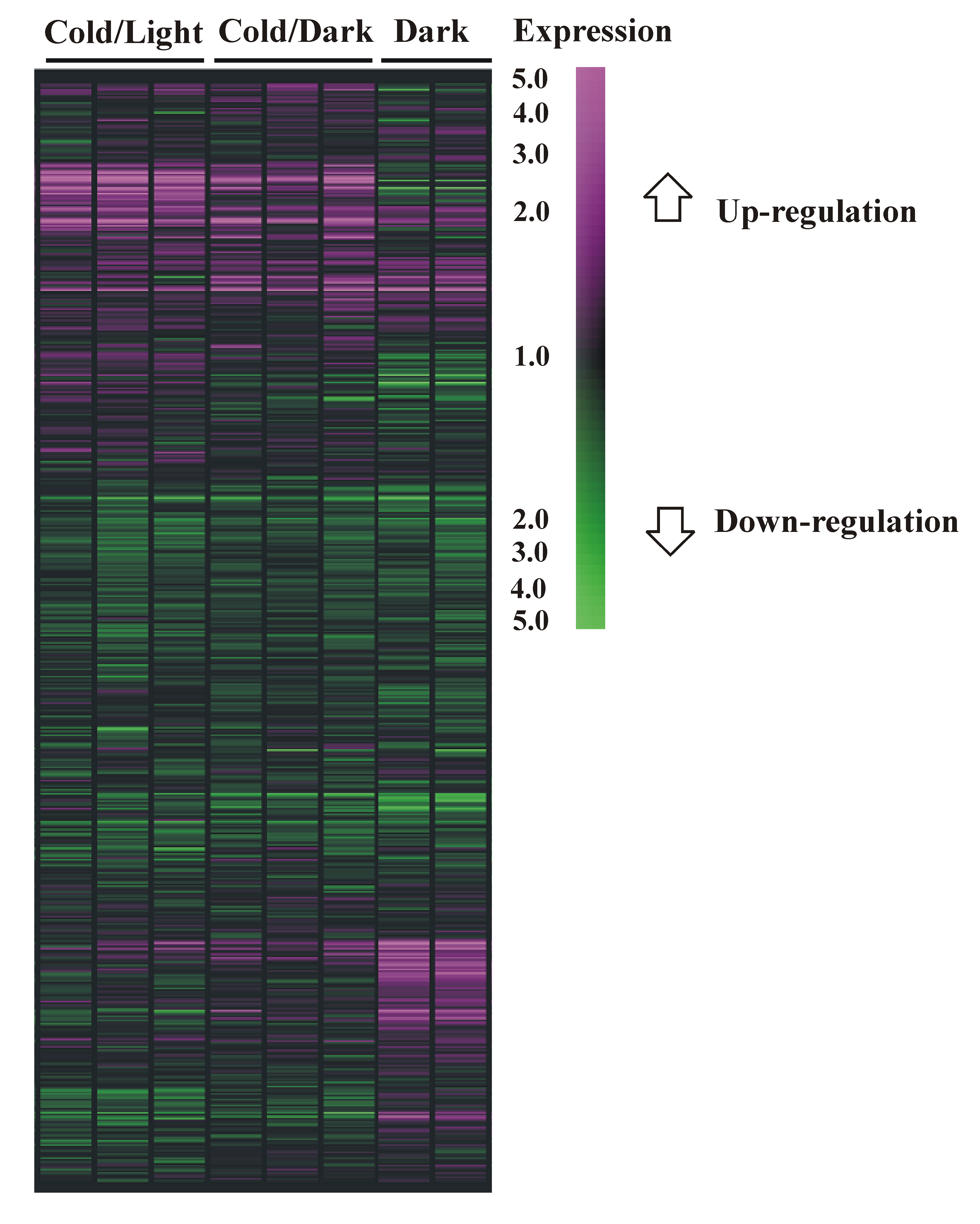

Supplement: Additional file 1 — Clustering of all genes under three different conditions (three biological replicates are shown for Cold/Light and Cold/Dark treatments and two for the Dark treatment). The brightness of colours indicates the expression level of the genes; magenta indicates up regulation and green down regulation of clustered genes. [file 1471-2229-8-13-S1.TIFF]
