# Supplementary material for: Light has a specific role in modulating Arabidopsis gene expression at low temperature
Source: BMC Plant Biol. 2008 Jan 29;8:13. doi: 10.1186/1471-2229-8-13 (PMC2253524; doi:10.1186/1471-2229-8-13)
Supplement: Additional file 6 — Visualising gene expression only under Cold/Light, Cold/Dark, Dark, conditions using MAPMAN analysis [91]. Following MAPMAN schemes were used: Metabolic Overview, Regulation Overview and Cellular Function Overview. Up regulated genes are shown in blue and down regulated genes in red. Visualized data is based on Additional files 3, 7 and 8. [file 1471-2229-8-13-S6.PDF]

Metabolic Overview: Only Cold/Light

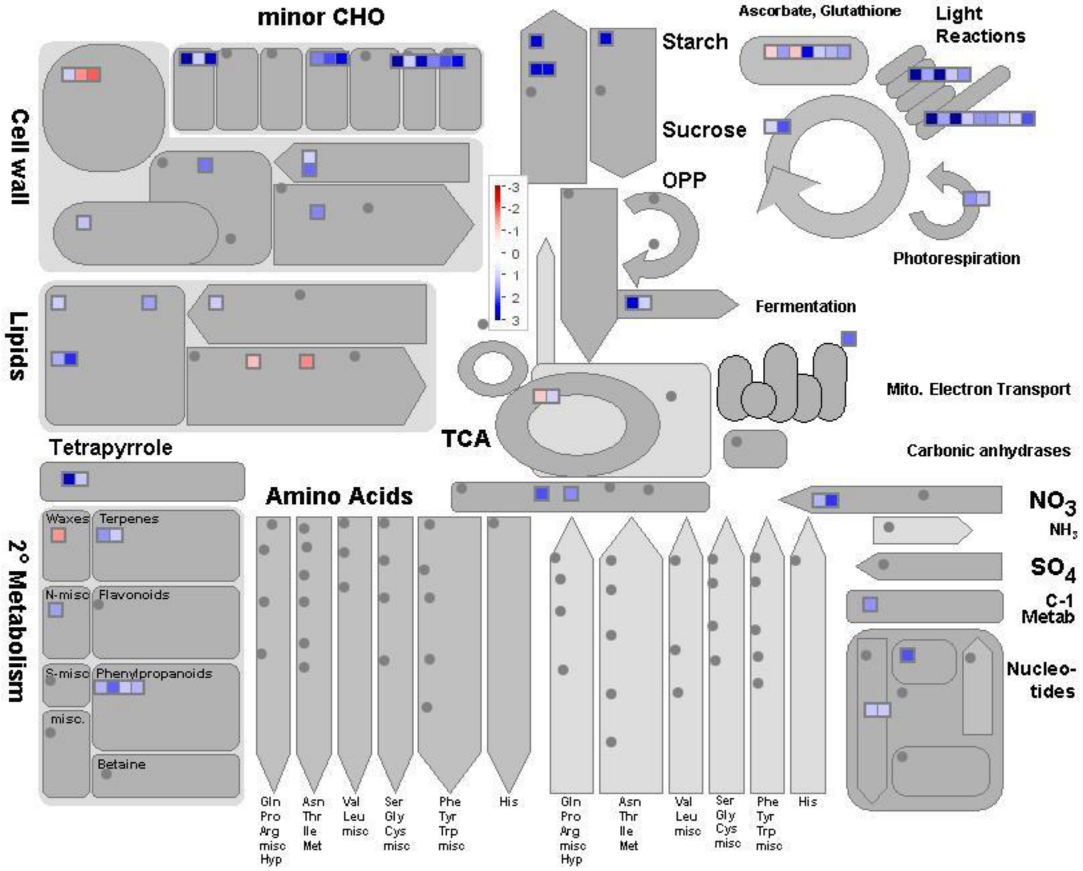

Wilcoxon Rank Sum Test | Log

Correction: Benjamini Hochberg corrected

Bin		Elements	Probability	Present
10.8	cell wall.pectin*esterases	3	0,67	shown
3	minor CHO metabolism	6	0,67	shown
2	major CHO metabolism	2	0,67	shown
3.4	minor CHO metabolism.myo-inositol	3	0,67	shown
11.9.3	lipid metabolism.lipid degradation.lysophospholipases	1	0,67	shown
16.7	secondary metabolism.wax	1	0,67	shown
1.1	PS.lightreaction	5	0,67	shown
8.1	TCA / org. transformation.TCA	2	0,67	shown
2.1.2	major CHO metabolism.synthesis.starch	1	0,67	shown
3.1	minor CHO metabolism.raffinose family	3	0,67	shown
2.2.2	major CHO metabolism.degradation.starch	1	0,67	shown
11.9.2	lipid metabolism.lipid degradation.lipases	1	0,71	shown
1	PS	9	0,71	shown
13.1.2	amino acid metabolism.synthesis.glutamate family	1	0,73	shown
23.3	nucleotide metabolism.salvage	1	0,73	shown
11.8	lipid metabolism.'exotics' (steroids, squalene etc)	2	0,77	shown
9	mitochondrial electron transport / ATP synthesis	1	0,77	shown
11.3	lipid metabolism.Phospholipid synthesis	1	0,77	shown
12.2	N-metabolism.ammonia metabolism	2	0,77	shown
23.1.2	nucleotide metabolism.synthesis.purine	2	0,77	shown
10.2	cell wall.cellulose synthesis	1	0,77	shown
19	tetrapyrrole synthesis	2	0,79	shown
11.2	lipid metabolism.FA desaturation	1	0,82	shown
10.6.2	cell wall.degradation.mannan-xylose-arabinose-fucose	1	0,86	shown
13.1.3	amino acid metabolism.synthesis.aspartate family	1	0,86	shown
21.2	redox.ascorbate and glutathione	7	0,89	shown
25	C1-metabolism	1	0,89	shown
5	fermentation	2	0,91	shown
10.7	cell wall.modification	1	0,91	shown
16.4	secondary metabolism.N misc	1	0,94	shown
11.4	lipid metabolism.TAG synthesis	1	0,95	shown
16.2	secondary metabolism.phenylpropanoids	4	0,95	shown
16.1	secondary metabolism.isoprenoids	2	0,96	shown
1.2	PS.photorespiration	2	0,96	shown
10.1	cell wall.precursor synthesis	2	0,97	shown
1.3	PS.calvin cyle	2	0,98	shown

Metabolic Overview: Only Cold/Dark

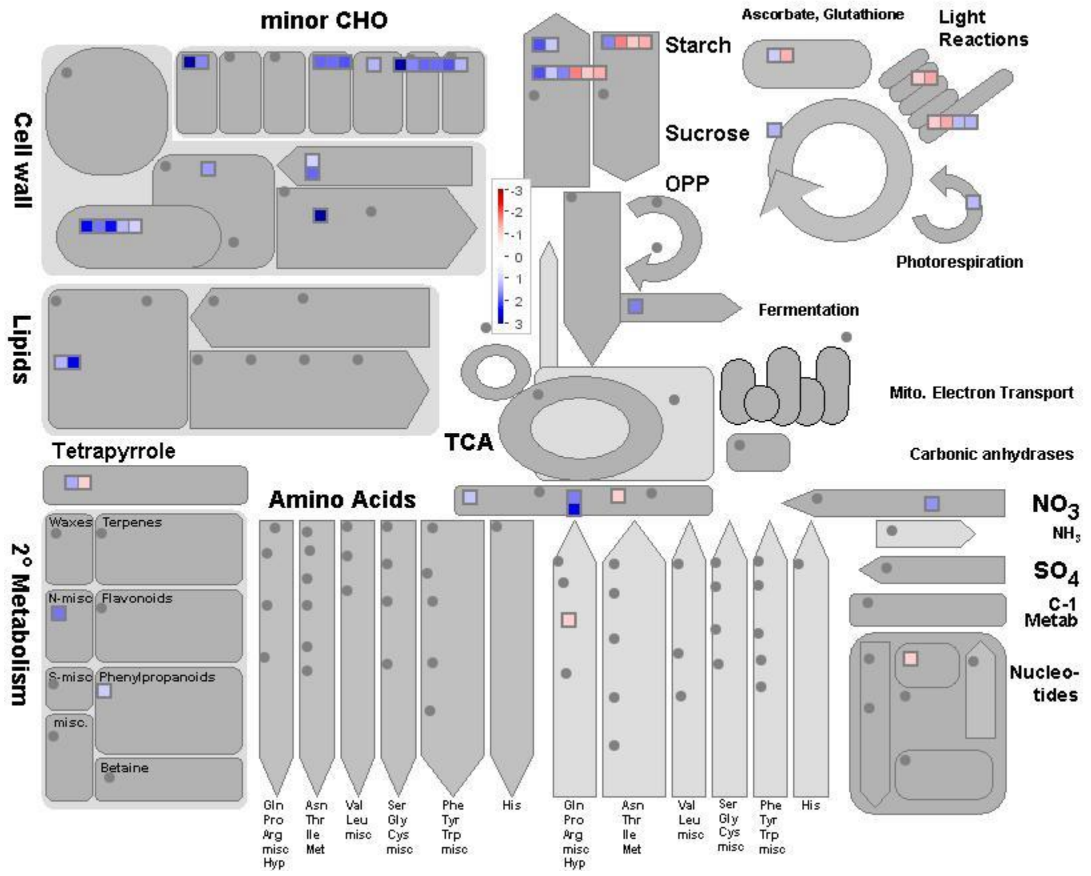

Wilcoxon Rank Sum Test | Log

Correction: Benjamini Hochberg corrected

Bin		Elements	Probability	Present
3	minor CHO metabolism	6	0,61	shown
1.1	PS.lightreaction	2	0,61	shown
2.2.2	major CHO metabolism.degradation.starch	4	0,61	shown
3.4	minor CHO metabolism.myo-inositol	3	0,61	shown
10.6.2	cell wall.degradation.mannan-xylose-arabinose-fucose	1	0,61	shown
21.2	redox.ascorbate and glutathione	2	0,61	shown
1	PS	4	0,61	shown
3.1	minor CHO metabolism.raffinose family	2	0,61	shown
13.1.3	amino acid metabolism.synthesis.aspartate family	2	0,61	shown
2	major CHO metabolism	6	0,61	shown
13.2.3.2	amino acid metabolism.degradation.aspartate family.thr...	1	0,61	shown
23.3	nucleotide metabolism.salvage	1	0,61	shown
13.1.4	amino acid metabolism.synthesis.branched chain group	1	0,61	shown
10.7	cell wall.modification	5	0,61	shown
11.8	lipid metabolism.'exotics' (steroids, squalene etc)	2	0,69	shown
16.2	secondary metabolism.phenylpropanoids	1	0,73	shown
19	tetrapyrrole synthesis	2	0,74	shown
16.4	secondary metabolism.N misc	1	0,74	shown
5	fermentation	1	0,79	shown
13.1.1	amino acid metabolism.synthesis.central amino acid m...	1	0,79	shown
2.1.2	major CHO metabolism.synthesis.starch	2	0,92	shown
1.2	PS.photorespiration	1	0,94	shown
12.1	N-metabolism.nitrate metabolism	1	0,94	shown
1.3	PS.calvin cyle	1	0,97	shown
3.5	minor CHO metabolism.others	1	0,97	shown
10.2	cell wall.cellulose synthesis	1	0,99	shown
10.1	cell wall.precursor synthesis	2	0,99	shown

Metabolic Overview: Dark

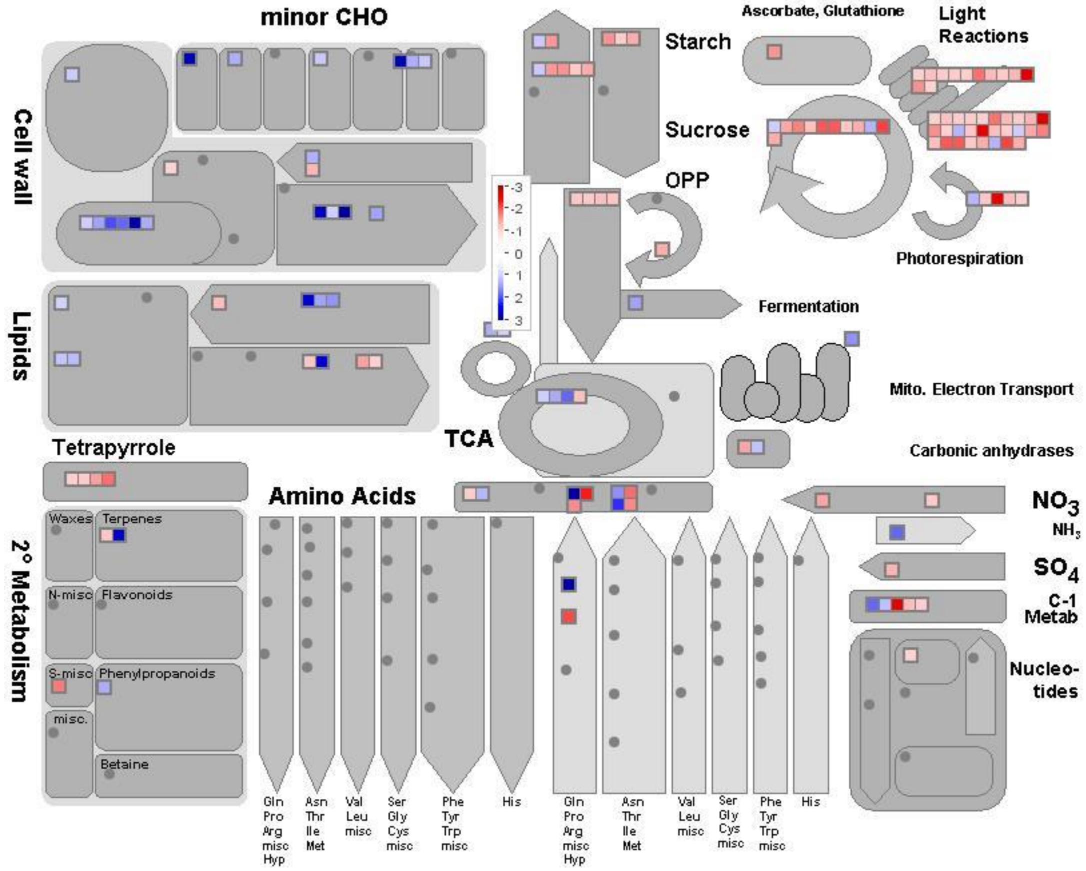

Wilcoxon Rank Sum Test | Log

Correction: Benjamini Hochberg corrected

Bin		Elements	Probability	Present
10.7	cell wall.modification	6	0,39	shown
11.1	lipid metabolism.FA synthesis and FA elongation	3	0,48	shown
10.6.2	cell wall.degradation.mannan-xylose-arabinose-fucose	3	0,48	shown
2	major CHO metabolism	5	0,48	shown
19	tetrapyrrole synthesis	4	0,48	shown
13.2.2.2	amino acid metabolism.degradation.glutamate family.pr...	1	0,48	shown
2.2.2	major CHO metabolism.degradation.starch	3	0,48	shown
3.1	minor CHO metabolism.raffinose family	1	0,48	shown
13.2.3.2	amino acid metabolism.degradation.aspartate family.thr...	1	0,48	shown
3	minor CHO metabolism	3	0,51	shown
16.5	secondary metabolism.sulfur-containing	1	0,51	shown
4	glycolysis	4	0,51	shown
1.2	PS.photorespiration	5	0,51	shown
21.2	redox.ascorbate and glutathione	1	0,54	shown
12.3	N-metabolism.N-degradation	1	0,54	shown
11.9.4	lipid metabolism.lipid degradation.beta-oxidation	2	0,58	shown
12.2	N-metabolism.ammonia metabolism	1	0,58	shown
7.2	OPP.non-reductive PP	1	0,62	shown
14	S-assimilation	1	0,65	shown
9	mitochondrial electron transport / ATP synthesis	1	0,67	shown
11.2	lipid metabolism.FA desaturation	1	0,73	shown
2.1.2	major CHO metabolism.synthesis.starch	2	0,73	shown
5	fermentation	1	0,73	shown
10.6.3	cell wall.degradation.pectate lyases and polygalacturon...	1	0,73	shown
16.2	secondary metabolism.phenylpropanoids	1	0,75	shown
13.1.3	amino acid metabolism.synthesis.aspartate family	3	0,75	shown
3.2	minor CHO metabolism.trehalose	1	0,75	shown
16.1	secondary metabolism.isoprenoids	2	0,75	shown
25	C1-metabolism	5	0,75	shown
8.3	TCA / org. transformation.carbonic anhydrases	2	0,75	shown
11.9.3	lipid metabolism.lipid degradation.lysophospholipases	2	0,75	shown
12.1	N-metabolism.nitrate metabolism	1	0,75	shown
8.1	TCA / org. transformation.TCA	4	0,77	shown
11.8	lipid metabolism.'exotics' (steroids, squalene etc)	2	0,81	shown
6	gluconeogenese/ glyoxylate cycle	2	0,86	shown
10.3	cell wall.hemicellulose synthesis	1	0,88	shown
23.3	nucleotide metabolism.salvage	1	0,88	shown
11.3	lipid metabolism.Phospholipid synthesis	1	0,89	shown
10.1	cell wall.precursor synthesis	2	0,89	shown
13.1.4	amino acid metabolism.synthesis.branched chain group	4	0,91	shown
3.4	minor CHO metabolism.myo-inositol	1	0,96	shown
13.1.1	amino acid metabolism.synthesis.central amino acid m...	2	0,99	shown
10.8	cell wall.pectin*esterases	1	1,00	shown
[illegible]	[illegible]	[illegible]	[illegible]	[illegible]

Regulation Overview: Only Cold/Light

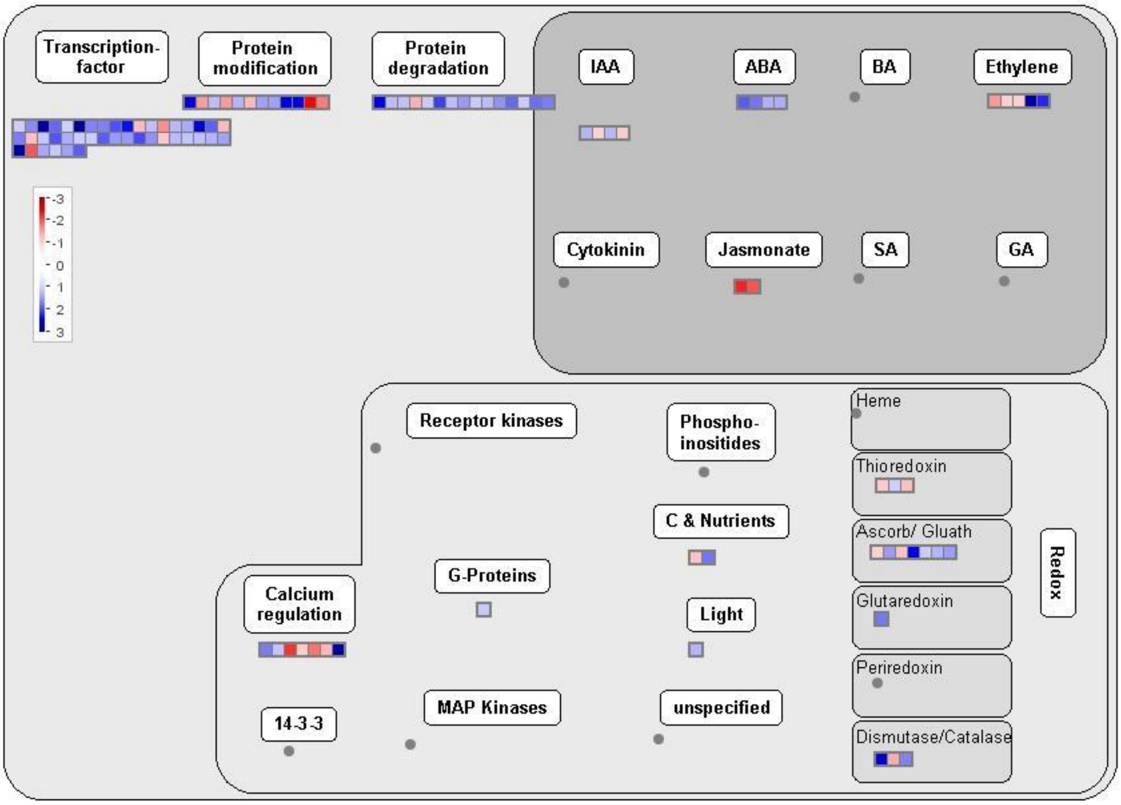

Wilcoxon Rank Sum Test | Log

Correction: Benjamini Hochberg corrected

Bin		Elements	Probability	Present
17.7	hormone metabolism.jasmonate	2	0,67	shown
21.1	redox.thioredoxin	3	0,67	shown
30.3	signalling.calcium	7	0,67	shown
17.2	hormone metabolism.auxin	4	0,71	shown
29.4	protein.postranslational modification	12	0,73	shown
17.1	hormone metabolism.abscisic acid	4	0,77	shown
27.3	RNA.regulation of transcription	42	0,77	shown
21.4	redox.glutaredoxins	1	0,80	shown
30.5	signalling.G-proteins	1	0,86	shown
21.2	redox.ascorbate and glutathione	7	0,89	shown
21.6	redox.dismutases and catalases	3	0,91	shown
30.1	signalling.in sugar and nutrient physiology	2	0,91	shown
17.5	hormone metabolism.ethylene	5	0,91	shown
29.5	protein degradation	15	0,91	shown
30.11	signalling.light	1	0,98	shown
10.8	cell wall.pectin*esterases	3	0,67	

Regulation Overview: Only Cold/Dark

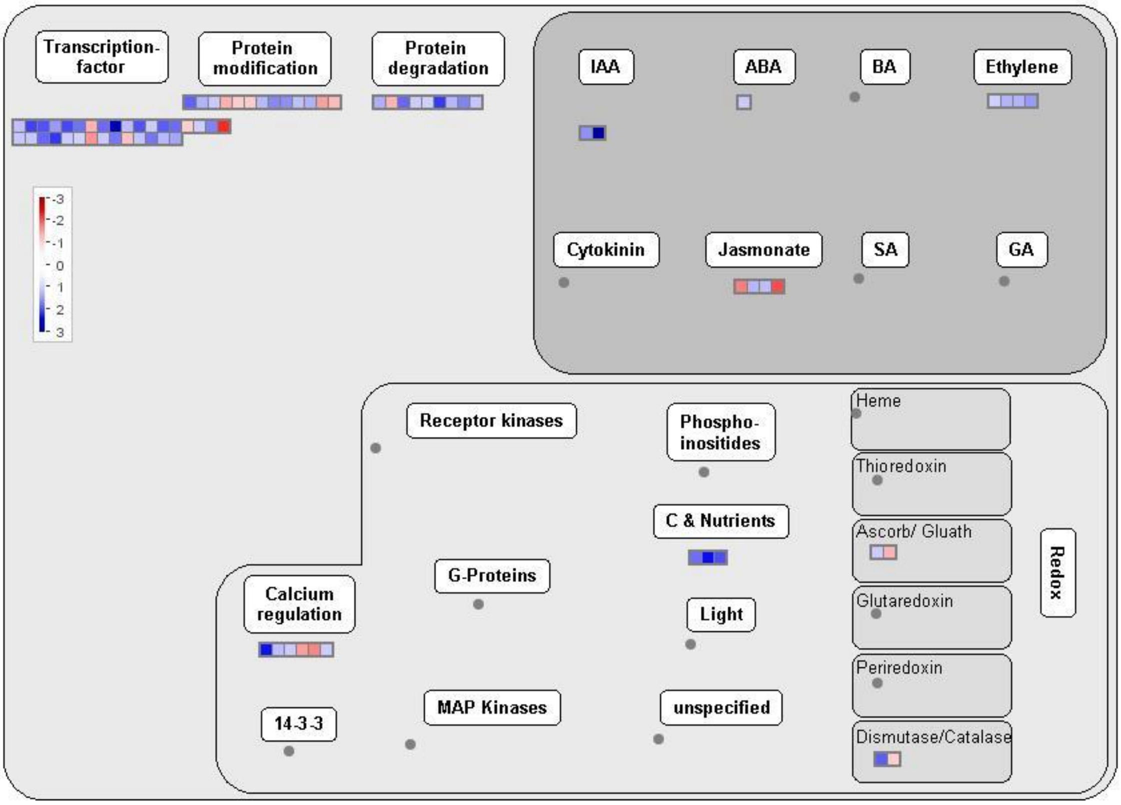

Wilcoxon Rank Sum Test | Log

Correction: Benjamini Hochberg corrected

Bin		Elements	Probability	Present
30.1	signalling.in sugar and nutrient ph...	3	0,61	shown
17.7	hormone metabolism.jasmonate	4	0,61	shown
29.4	protein.postranslational modification	13	0,61	shown
21.2	redox.ascorbate and glutathione	2	0,61	shown
30.3	signalling.calcium	6	0,61	shown
17.2	hormone metabolism.auxin	2	0,61	shown
17.1	hormone metabolism.abscisic acid	1	0,74	shown
17.5	hormone metabolism.ethylene	4	0,79	shown
29.5	protein degradation	9	0,88	shown
21.6	redox.dismutases and catalases	2	0,99	shown
27.3	RNA.regulation of transcription	32	0,99	shown

Regulation Overview: Dark

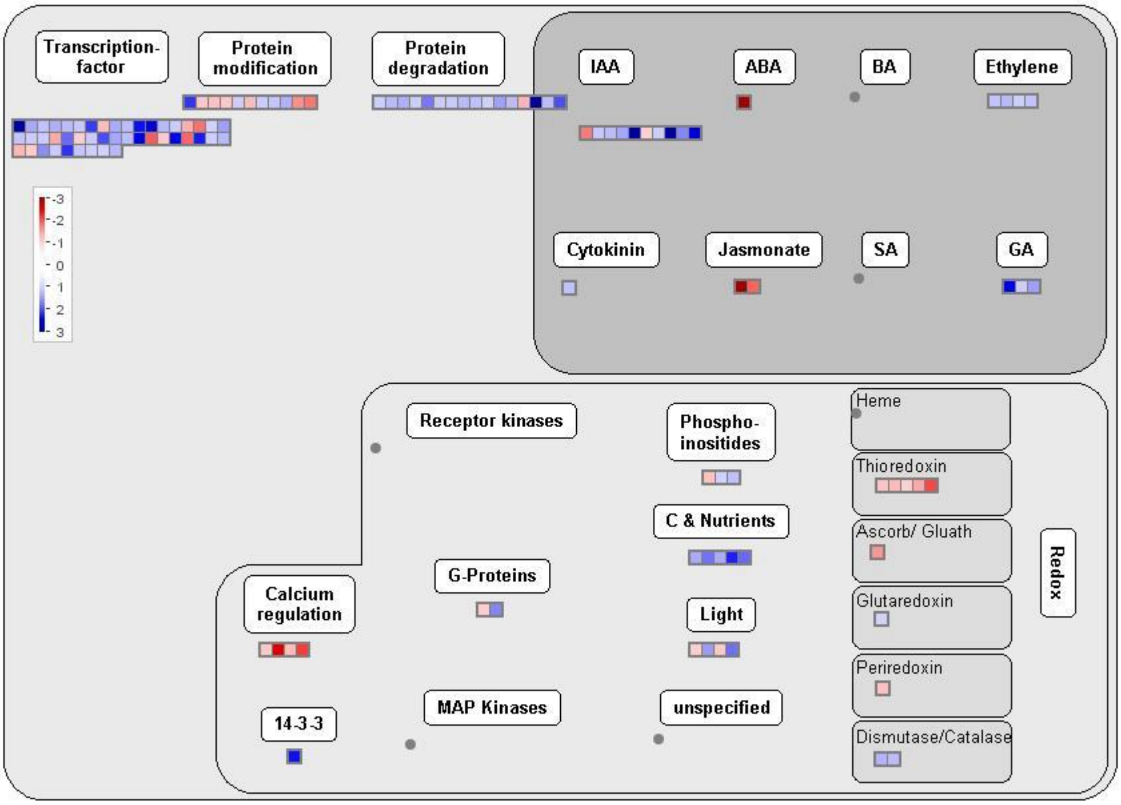

Wilcoxon Rank Sum Test | Log

Correction: Benjamini Hochberg corrected

Bin		Elements	Probability	Present
30.1	signalling.in sugar and nutrient ph...	5	0,39	shown
30.3	signalling.calcium	4	0,39	shown
17.7	hormone metabolism.jasmonate	2	0,39	shown
21.1	redox.thioredoxin	5	0,48	shown
27.3	RNA.regulation of transcription	45	0,48	shown
17.2	hormone metabolism.auxin	10	0,48	shown
17.1	hormone metabolism.abscisic acid	1	0,48	shown
29.5	protein.degradation	16	0,48	shown
30.7	signalling.14-3-3 proteins	1	0,48	shown
17.6	hormone metabolism.gibberelin	3	0,52	shown
21.2	redox.ascorbate and glutathione	1	0,54	shown
29.4	protein.postranslational modification	11	0,62	shown
21.5	redox.periredoxins	1	0,74	shown
21.6	redox.dismutases and catalases	2	0,75	shown
30.11	signalling.light	4	0,78	shown
30.5	signalling.G-proteins	2	0,84	shown
17.5	hormone metabolism.ethylene	4	0,84	shown
30.4	signalling.phosphinositides	3	0,86	shown
17.4	hormone metabolism.cytokinin	1	0,88	shown
21.4	redox.glutaredoxins	1	0,88	shown

Cellular Function Overview: Only Cold/Light

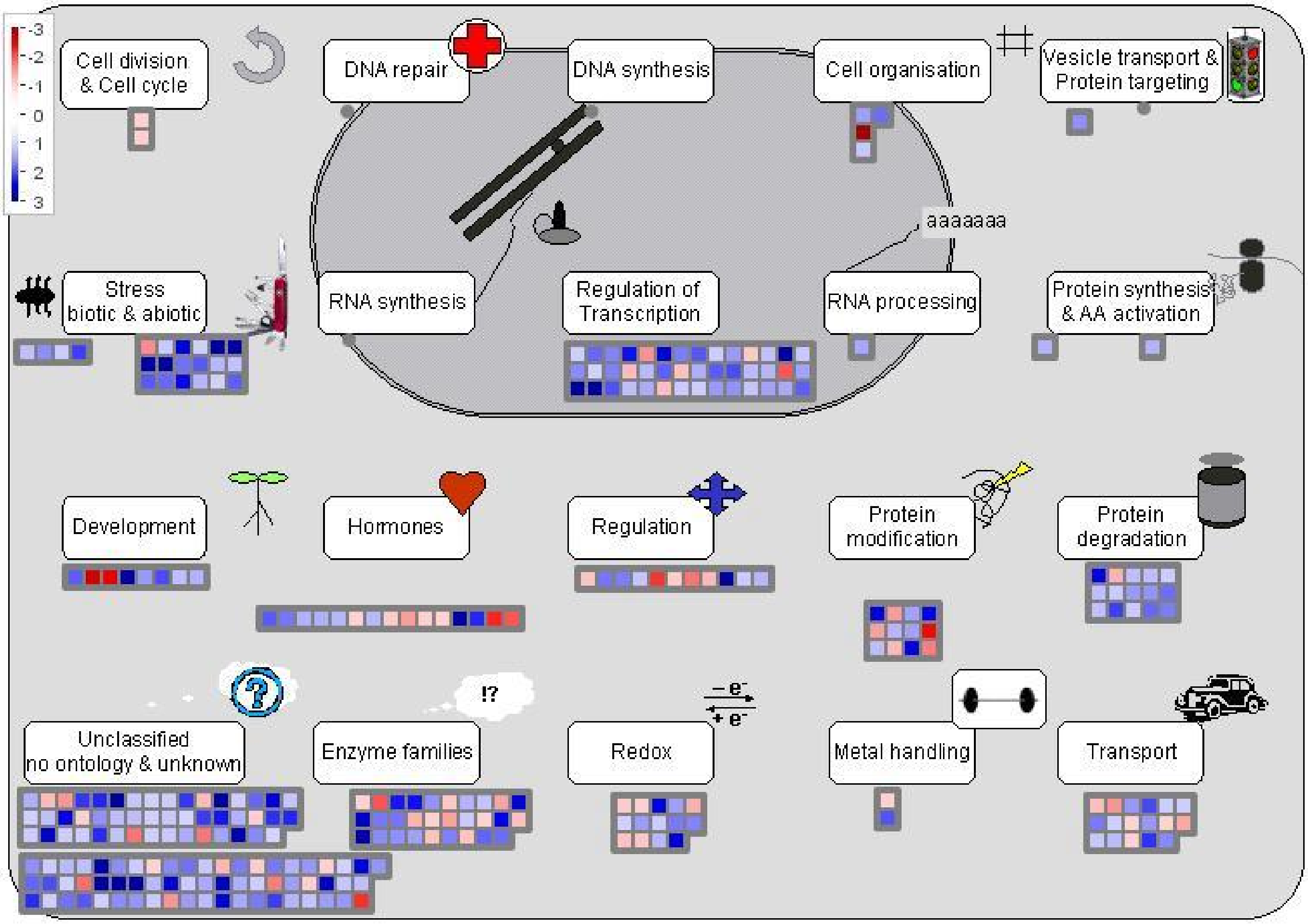

Wilcoxon Rank Sum Test | Log

Correction: Benjamini Hochberg corrected

Bin		Elements	Probability	Present
20.2	stress.abiotic	18	0,67	shown
30	signalling	11	0,67	shown
31.3	cell.cycle	2	0,67	shown
34	transport	17	0,67	shown
17	hormone metabolism	15	0,71	shown
35.2	not assigned.unknown	61	0,73	shown
29.4	protein.postranslational modification	12	0,73	shown
21	redox.regulation	14	0,77	shown
27.3	RNA.regulation of transcription	42	0,77	shown
31.1	cell.organisation	4	0,86	shown
31.4	cell. vesicle transport	1	0,89	shown
20.1	stress.biotic	4	0,91	shown
29.5	protein degradation	15	0,91	shown
26	misc	29	0,94	shown
27.1	RNA.processing	1	0,96	shown
33	development	8	0,96	shown
35.1	not assigned.no ontology	47	0,97	shown
15	metal handling	2	0,97	shown
29.1	protein.aa activation	1	0,98	shown

Cellular Function Overview: Only Cold/Dark

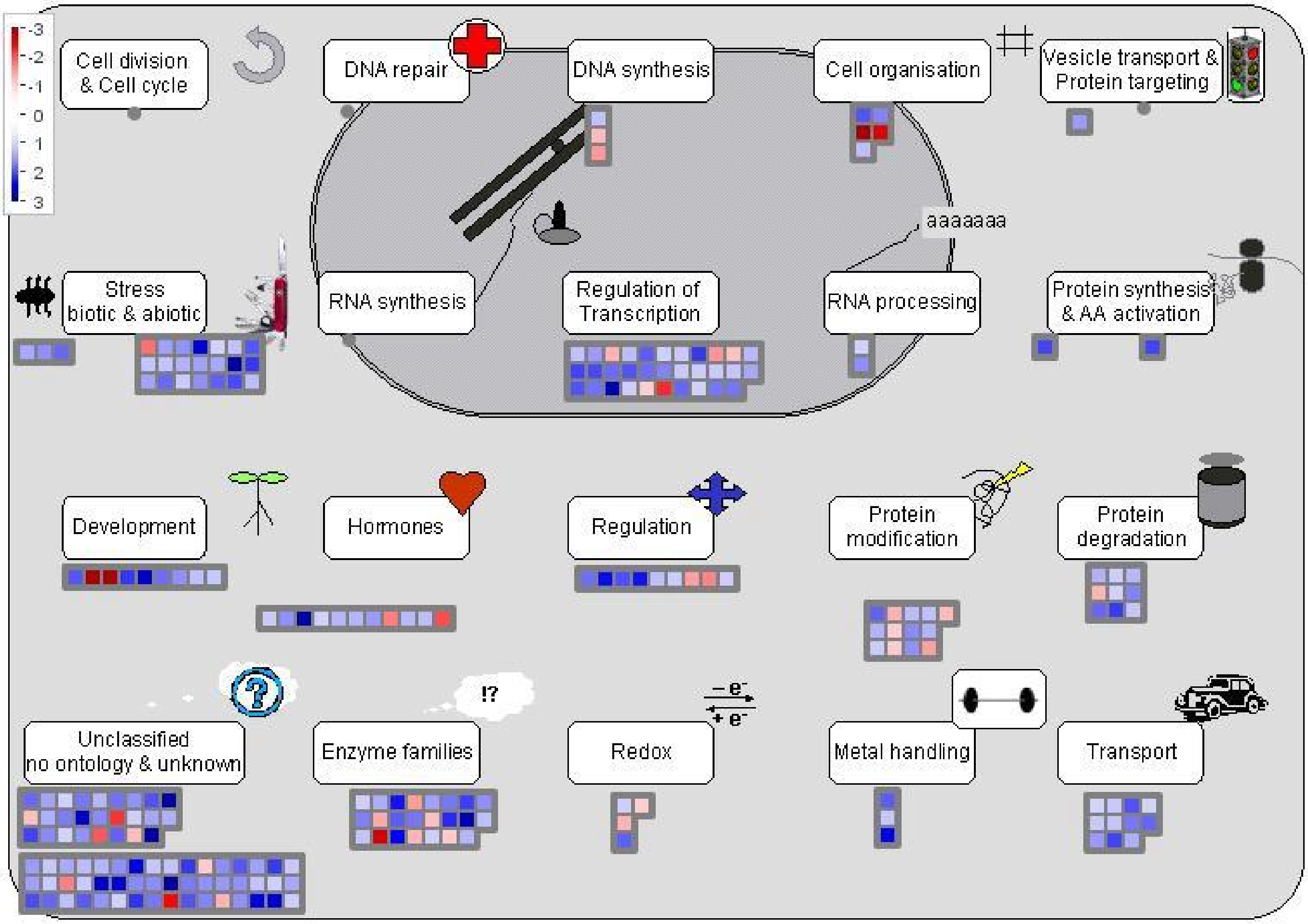

Wilcoxon Rank Sum Test | Log

Correction: Benjamini Hochberg corrected

Bin		Elements	Probability	Present
35.2	not assigned.unknown	48	0,61	shown
28.1	DNA.synthesis/chromatin structure	3	0,61	shown
29.4	protein.postranslational modification	13	0,61	shown
29.1	protein.aa activation	1	0,61	shown
21	redox.regulation	4	0,61	shown
17	hormone metabolism	11	0,61	shown
15	metal handling	3	0,61	shown
20.2	stress.abiotic	21	0,68	shown
31.1	cell.organisation	5	0,70	shown
35.1	not assigned.no ontology	26	0,74	shown
20.1	stress.biotic	3	0,74	shown
29.5	protein degradation	9	0,88	shown
27.1	RNA.processing	2	0,89	shown
33	development	9	0,94	shown
31.4	cell. vesicle transport	1	0,98	shown
26	misc	23	0,99	shown
30	signalling	9	0,99	shown
27.3	RNA.regulation of transcription	32	0,99	shown
34	transport	11	0,99	shown

Cellular Function Overview: Dark

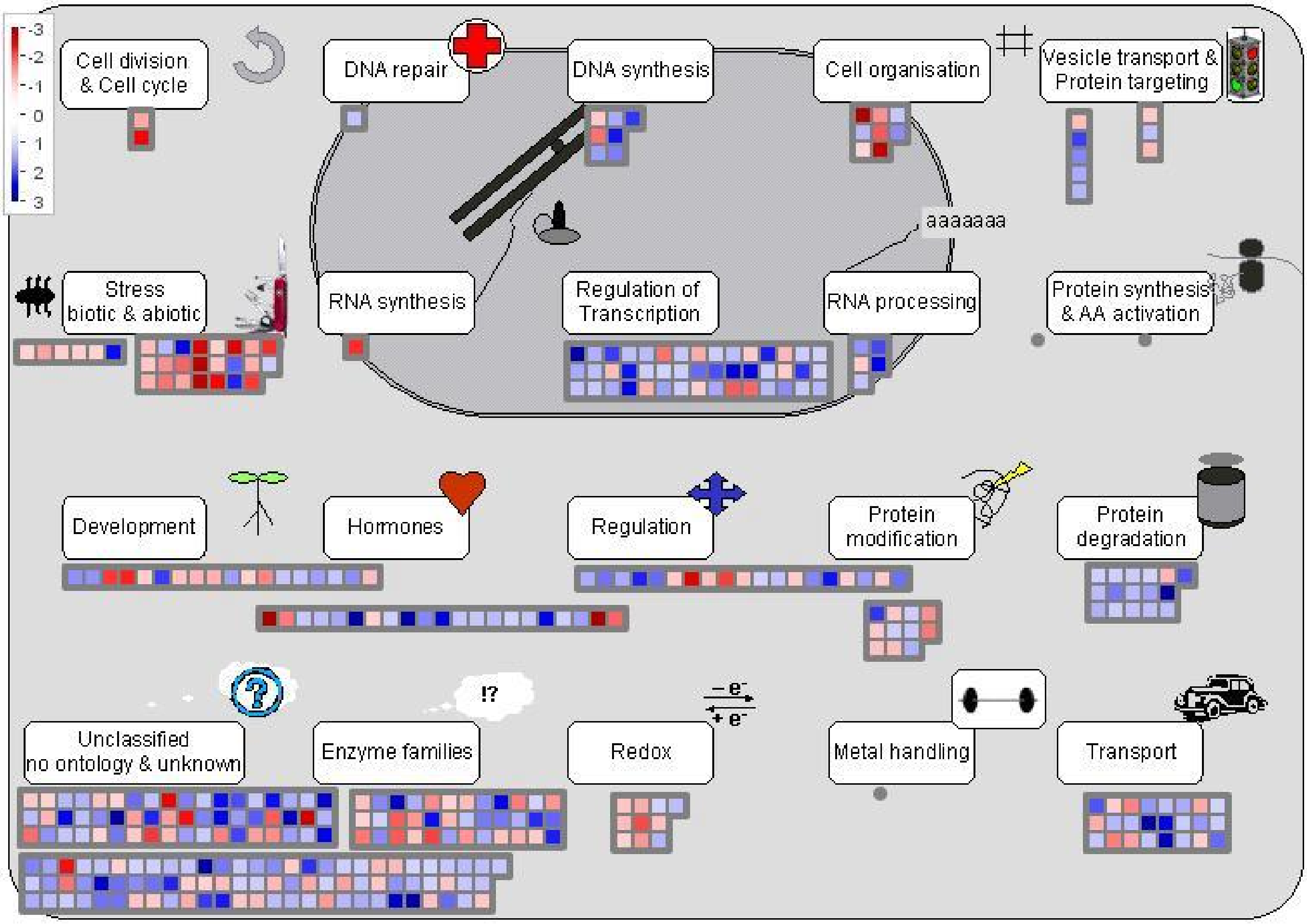

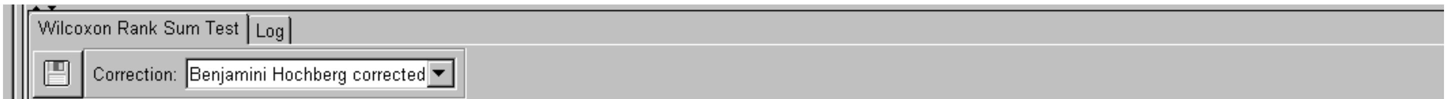

Bin		Elements	Probability	Present
20.2	stress.abiotic	23	1,75E-2	shown
35.2	not assigned.unknown	82	0,39	shown
31.3	cell.cycle	2	0,48	shown
21	redox.regulation	10	0,48	shown
27.3	RNA.regulation of transcription	45	0,48	shown
31.1	cell.organisation	8	0,48	shown
29.5	protein degradation	16	0,48	shown
35.1	not assigned.no ontology	54	0,48	shown
27.2	RNA.transcription	1	0,48	shown
27.1	RNA.processing	5	0,48	shown
28.1	DNA.synthesis/chromatin structure	7	0,52	shown
31.4	cell. vesicle transport	5	0,54	shown
29.4	protein.postranslational modification	11	0,62	shown
34	transport	24	0,65	shown
17	hormone metabolism	21	0,73	shown
30	signalling	19	0,75	shown
29.3	protein.targeting	3	0,75	shown
33	development	18	0,77	shown
20.1	stress.biotic	6	0,81	shown
26	misc	36	0,86	shown
28.2	DNA.repair	1	0,89	shown
1	PS	28	2,93E-3	[illegible]
